# Supplementary material for: Neuropeptide F regulates courtship in Drosophila through a male-specific neuronal circuit
Source: eLife. 2019 Aug 12;8:e49574. doi: 10.7554/eLife.49574 (PMC6721794; doi:10.7554/eLife.49574)
Supplement: Figure 1—source data 2. [file elife-49574-fig1-data2.docx]

Figure 1A summary statistics

|  | UAS-Shibire/+ @23c | UAS-Shibire/+ @31c | npf-Gal4/+ @23c | npf-Gal4/+ @31c | UAS-Shibire/#25681 @23c | UAS-Shibire/#25681 @31c |
| --- | --- | --- | --- | --- | --- | --- |
| Number of values | 4 | 4 | 4 | 4 | 4 | 4 |
|  |  |  |  |  |  |  |
| Minimum | 0.0 | 0.0 | 0.0 | 0.0 | 0.0 | 66.67 |
| 25% Percentile | 0.0 | 0.0 | 0.0 | 0.0 | 0.0 | 66.67 |
| Median | 8.333 | 8.333 | 0.0 | 0.0 | 0.0 | 66.67 |
| 75% Percentile | 16.67 | 29.17 | 12.50 | 12.50 | 25.00 | 79.17 |
| Maximum | 16.67 | 33.33 | 16.67 | 16.67 | 33.33 | 83.33 |
|  |  |  |  |  |  |  |
| Mean | 8.333 | 12.50 | 4.167 | 4.167 | 8.333 | 70.83 |
| Std. Deviation | 9.623 | 15.96 | 8.333 | 8.333 | 16.67 | 8.333 |
| Std. Error | 4.811 | 7.979 | 4.167 | 4.167 | 8.333 | 4.167 |
|  |  |  |  |  |  |  |
| Lower 95% CI of mean | -6.978 | -12.89 | -9.094 | -9.094 | -18.19 | 57.57 |
| Upper 95% CI of mean | 23.64 | 37.89 | 17.43 | 17.43 | 34.85 | 84.09 |
|  |  |  |  |  |  |  |
| Sum | 33.33 | 50.00 | 16.67 | 16.67 | 33.33 | 283.3 |

| Parameter |  |
| --- | --- |
| Table Analyzed | Percentage Initiate Courtship 6x4 |
| Column E | UAS-Shibire/#25681 @23c |
| vs | vs |
| Column F | UAS-Shibire/#25681 @31c |
|  |  |
| Mann Whitney test |  |
| P value | 0.0265 |
| Exact or approximate P value? | Gaussian Approximation |
| P value summary | * |
| Are medians signif. different? (P < 0.05) | Yes |
| One- or two-tailed P value? | Two-tailed |
| Sum of ranks in column E,F | 10 , 26 |
| Mann-Whitney U | 0.0000 |

Figure 1B summary statistics

|  | UAS-Shibire/+ @23c | UAS-Shibire/+ @31c | npf-Gal4/+ @23c | npf-Gal4/+ @31c | UAS-Shibire/#25681 @23c | UAS-Shibire/#25681 @31c |
| --- | --- | --- | --- | --- | --- | --- |
| Number of values | 24 | 24 | 24 | 24 | 24 | 24 |
|  |  |  |  |  |  |  |
| Minimum | 0.0 | 0.0 | 0.0 | 0.0 | 0.0 | 0.0 |
| 25% Percentile | 0.0 | 0.0 | 0.0 | 0.0 | 0.0 | 0.0 |
| Median | 0.0 | 0.0 | 0.0 | 0.0 | 0.0 | 0.06833 |
| 75% Percentile | 0.0 | 0.0 | 0.0 | 0.0 | 0.0 | 0.2025 |
| Maximum | 0.1000 | 0.0800 | 0.0800 | 0.0500 | 0.1200 | 0.9000 |
|  |  |  |  |  |  |  |
| Mean | 0.00625 | 0.0075 | 0.003333 | 0.002083 | 0.007083 | 0.1886 |
| Std. Deviation | 0.02242 | 0.02172 | 0.01633 | 0.01021 | 0.02612 | 0.2907 |
| Std. Error | 0.004577 | 0.004433 | 0.003333 | 0.002083 | 0.005332 | 0.05933 |
|  |  |  |  |  |  |  |
| Lower 95% CI of mean | -0.003218 | -0.001671 | -0.003562 | -0.002226 | -0.003947 | 0.06588 |
| Upper 95% CI of mean | 0.01572 | 0.01667 | 0.01023 | 0.006393 | 0.01811 | 0.3113 |
|  |  |  |  |  |  |  |
| Sum | 0.1500 | 0.1800 | 0.0800 | 0.0500 | 0.1700 | 4.527 |

| Parameter |  |
| --- | --- |
| Table Analyzed | Preheat 20' CI |
| Column E | UAS-Shibire/#25681 @23c |
| vs | vs |
| Column F | UAS-Shibire/#25681 @31c |
|  |  |
| Mann Whitney test |  |
| P value | < 0.0001 |
| Exact or approximate P value? | Gaussian Approximation |
| P value summary | *** |
| Are medians signif. different? (P < 0.05) | Yes |
| One- or two-tailed P value? | Two-tailed |
| Sum of ranks in column E,F | 402.5 , 773.5 |
| Mann-Whitney U | 102.5 |

Figure 1C summary statistics

|  | UAS-Shibire/+ @23c | UAS-Shibire/+ @31c | npf-Gal4/+ @23c | npf-Gal4/+ @31c | UAS-Shibire/#25681 @23c | UAS-Shibire/#25681 @31c |
| --- | --- | --- | --- | --- | --- | --- |
| Number of values | 6 | 6 | 6 | 6 | 6 | 6 |
|  |  |  |  |  |  |  |
| Minimum | 0.0 | 0.0 | 0.0 | 0.0 | 0.0 | 0.2500 |
| 25% Percentile | 0.0 | 0.0 | 0.0 | 0.0 | 0.0 | 0.2725 |
| Median | 0.0 | 0.0050 | 0.0 | 0.0 | 0.0165 | 0.3150 |
| 75% Percentile | 0.0275 | 0.0250 | 0.0125 | 0.0325 | 0.03125 | 0.3625 |
| Maximum | 0.0500 | 0.0400 | 0.0200 | 0.0400 | 0.0500 | 0.4000 |
|  |  |  |  |  |  |  |
| Mean | 0.01167 | 0.01167 | 0.0050 | 0.01167 | 0.0180 | 0.3183 |
| Std. Deviation | 0.02041 | 0.01602 | 0.008367 | 0.01835 | 0.01903 | 0.05345 |
| Std. Error | 0.008333 | 0.006540 | 0.003416 | 0.007491 | 0.007767 | 0.02182 |
|  |  |  |  |  |  |  |
| Lower 95% CI of mean | -0.009755 | -0.005146 | -0.003780 | -0.007589 | -0.001967 | 0.2622 |
| Upper 95% CI of mean | 0.03309 | 0.02848 | 0.01378 | 0.03092 | 0.03797 | 0.3744 |
|  |  |  |  |  |  |  |
| Sum | 0.0700 | 0.0700 | 0.0300 | 0.0700 | 0.1080 | 1.910 |

| Parameter |  |
| --- | --- |
| Table Analyzed | MM Chaining Index |
| Column E | UAS-Shibire/#25681 @23c |
| vs | vs |
| Column F | UAS-Shibire/#25681 @31c |
|  |  |
| Mann Whitney test |  |
| P value | 0.0050 |
| Exact or approximate P value? | Gaussian Approximation |
| P value summary | ** |
| Are medians signif. different? (P < 0.05) | Yes |
| One- or two-tailed P value? | Two-tailed |
| Sum of ranks in column E,F | 21 , 57 |
| Mann-Whitney U | 0.0000 |
