# Supplementary material for: Neuropeptide F regulates courtship in Drosophila through a male-specific neuronal circuit
Source: eLife. 2019 Aug 12;8:e49574. doi: 10.7554/eLife.49574 (PMC6721794; doi:10.7554/eLife.49574)
Supplement: Figure 1—source data 4. [file elife-49574-fig1-data4.docx]

Figure 1F

|  | npf[LexA]/npf[1] | npf[GR];npf[LexA] | w+;w1118 |
| --- | --- | --- | --- |
| Number of values | 24 | 24 | 24 |
|  |  |  |  |
| Minimum | 0.0 | 0.0 | 0.0 |
| 25% Percentile | 0.1050 | 0.0 | 0.0 |
| Median | 0.3000 | 0.0 | 0.0 |
| 75% Percentile | 0.5725 | 0.1000 | 0.1900 |
| Maximum | 0.9000 | 0.7300 | 0.7200 |
|  |  |  |  |
| Mean | 0.3496 | 0.1208 | 0.1021 |
| Std. Deviation | 0.2594 | 0.2390 | 0.1851 |
| Std. Error | 0.05295 | 0.04878 | 0.03778 |
|  |  |  |  |
| Lower 95% CI of mean | 0.2400 | 0.01993 | 0.02393 |
| Upper 95% CI of mean | 0.4591 | 0.2217 | 0.1802 |
|  |  |  |  |
| Sum | 8.390 | 2.900 | 2.450 |

| Parameter |  |  |  |  |
| --- | --- | --- | --- | --- |
| Table Analyzed | MF Grouped vs mature active |  |  |  |
|  |  |  |  |  |
| Kruskal-Wallis test |  |  |  |  |
| P value | < 0.0001 |  |  |  |
| Exact or approximate P value? | Gaussian Approximation |  |  |  |
| P value summary | *** |  |  |  |
| Do the medians vary signif. (P < 0.05) | Yes |  |  |  |
| Number of groups | 3 |  |  |  |
| Kruskal-Wallis statistic | 19.03 |  |  |  |
|  |  |  |  |  |
| Dunn's Multiple Comparison Test | Difference in rank sum | Significant? P < 0.05? | Summary |  |
| npf[LexA]/npf[1] vs npf[GR];npf[LexA] | 21.46 | Yes | *** |  |
| npf[LexA]/npf[1] vs w+;w1118 | 21.23 | Yes | *** |  |
| npf[GR];npf[LexA] vs w+;w1118 | -0.2292 | No | ns |  |

Figure 1G

|  | w+;w1118 | npf[LexA]/npf[1] | npf[GR];npf[LexA] |
| --- | --- | --- | --- |
| Number of values | 12 | 12 | 12 |
|  |  |  |  |
| Minimum | 0.0 | 0.2000 | 0.0 |
| 25% Percentile | 0.0 | 0.4000 | 0.0 |
| Median | 0.1500 | 0.5850 | 0.0250 |
| 75% Percentile | 0.4625 | 0.7375 | 0.3975 |
| Maximum | 0.5000 | 0.8000 | 0.5500 |
|  |  |  |  |
| Mean | 0.2133 | 0.5533 | 0.1567 |
| Std. Deviation | 0.2155 | 0.2058 | 0.2127 |
| Std. Error | 0.06221 | 0.05942 | 0.06141 |
|  |  |  |  |
| Lower 95% CI of mean | 0.07641 | 0.4226 | 0.02150 |
| Upper 95% CI of mean | 0.3503 | 0.6841 | 0.2918 |
|  |  |  |  |
| Sum | 2.560 | 6.640 | 1.880 |

| Parameter |  |  |  |  |
| --- | --- | --- | --- | --- |
| Table Analyzed | MF Isolated vs simulans |  |  |  |
|  |  |  |  |  |
| Kruskal-Wallis test |  |  |  |  |
| P value | 0.0007 |  |  |  |
| Exact or approximate P value? | Gaussian Approximation |  |  |  |
| P value summary | *** |  |  |  |
| Do the medians vary signif. (P < 0.05) | Yes |  |  |  |
| Number of groups | 3 |  |  |  |
| Kruskal-Wallis statistic | 14.42 |  |  |  |
|  |  |  |  |  |
| Dunn's Multiple Comparison Test | Difference in rank sum | Significant? P < 0.05? | Summary |  |
| w+;w1118 vs npf[LexA]/npf[1] | -12.54 | Yes | ** |  |
| w+;w1118 vs npf[GR];npf[LexA] | 2.542 | No | ns |  |
| npf[LexA]/npf[1] vs npf[GR];npf[LexA] | 15.08 | Yes | ** |  |

Figure 1H

|  | npf[LexA]/npf[1] | w+;w1118 | npf[GR];npf[LexA] |
| --- | --- | --- | --- |
| Number of values | 19 | 19 | 24 |
|  |  |  |  |
| 25% Percentile | 0.1400 | 0.0 | 0.0 |
| Median | 0.2300 | 0.0 | 0.0 |
| 75% Percentile | 0.5500 | 0.0500 | 0.0 |
|  |  |  |  |
| Mean | 0.2895 | 0.03053 | 0.0550 |
| Std. Deviation | 0.2026 | 0.05462 | 0.1441 |
| Std. Error | 0.04649 | 0.01253 | 0.02941 |
|  |  |  |  |
| Lower 95% CI of mean | 0.1918 | 0.004202 | -0.005848 |
| Upper 95% CI of mean | 0.3871 | 0.05685 | 0.1158 |
|  |  |  |  |
| Sum | 5.500 | 0.5800 | 1.320 |

| Parameter |  |  |  |  |
| --- | --- | --- | --- | --- |
| Table Analyzed | MM Isolated vs grouped male |  |  |  |
|  |  |  |  |  |
| Kruskal-Wallis test |  |  |  |  |
| P value | < 0.0001 |  |  |  |
| Exact or approximate P value? | Gaussian Approximation |  |  |  |
| P value summary | *** |  |  |  |
| Do the medians vary signif. (P < 0.05) | Yes |  |  |  |
| Number of groups | 3 |  |  |  |
| Kruskal-Wallis statistic | 32.59 |  |  |  |
|  |  |  |  |  |
| Dunn's Multiple Comparison Test | Difference in rank sum | Significant? P < 0.05? | Summary |  |
| npf[LexA]/npf[1] vs w+;w1118 | 25.82 | Yes | *** |  |
| npf[LexA]/npf[1] vs npf[GR];npf[LexA] | 26.74 | Yes | *** |  |
| w+;w1118 vs npf[GR];npf[LexA] | 0.9265 | No | ns |  |

Figure 1I

|  | w+;w1118 | npf[LexA]/npf[1] | npf[GR];npf[LexA] |
| --- | --- | --- | --- |
| Number of values | 12 | 12 | 12 |
|  |  |  |  |
| 25% Percentile | 1.000 | 0.8625 | 0.9250 |
| Median | 1.000 | 1.000 | 1.000 |
| 75% Percentile | 1.000 | 1.000 | 1.000 |
|  |  |  |  |
| Mean | 0.9583 | 0.9417 | 0.9542 |
| Std. Deviation | 0.1018 | 0.09311 | 0.08908 |
| Std. Error | 0.02938 | 0.02688 | 0.02572 |
|  |  |  |  |
| Lower 95% CI of mean | 0.8937 | 0.8825 | 0.8976 |
| Upper 95% CI of mean | 1.023 | 1.001 | 1.011 |
|  |  |  |  |
| Sum | 11.50 | 11.30 | 11.45 |

| Parameter |  |  |  |  |
| --- | --- | --- | --- | --- |
| Table Analyzed | MF Preference |  |  |  |
|  |  |  |  |  |
| Kruskal-Wallis test |  |  |  |  |
| P value | 0.7392 |  |  |  |
| Exact or approximate P value? | Gaussian Approximation |  |  |  |
| P value summary | ns |  |  |  |
| Do the medians vary signif. (P < 0.05) | No |  |  |  |
| Number of groups | 3 |  |  |  |
| Kruskal-Wallis statistic | 0.6044 |  |  |  |

Figure 1J

|  | npf[LexA]/npf[1] | w+;w1118 | npf[GR];npf[LexA] |
| --- | --- | --- | --- |
| Number of values | 24 | 24 | 24 |
|  |  |  |  |
| 25% Percentile | 0.2000 | 0.0 | 0.0 |
| Median | 0.3550 | 0.0 | 0.0 |
| 75% Percentile | 0.5975 | 0.1925 | 0.2600 |
|  |  |  |  |
| Mean | 0.3975 | 0.1175 | 0.1446 |
| Std. Deviation | 0.2535 | 0.2044 | 0.2648 |
| Std. Error | 0.05175 | 0.04172 | 0.05405 |
|  |  |  |  |
| Lower 95% CI of mean | 0.2904 | 0.03119 | 0.03277 |
| Upper 95% CI of mean | 0.5046 | 0.2038 | 0.2564 |
|  |  |  |  |
| Sum | 9.540 | 2.820 | 3.470 |

| Parameter |  |  |  |  |
| --- | --- | --- | --- | --- |
| Table Analyzed | MF Grouped vs new-eclosion |  |  |  |
|  |  |  |  |  |
| Kruskal-Wallis test |  |  |  |  |
| P value | < 0.0001 |  |  |  |
| Exact or approximate P value? | Gaussian Approximation |  |  |  |
| P value summary | *** |  |  |  |
| Do the medians vary signif. (P < 0.05) | Yes |  |  |  |
| Number of groups | 3 |  |  |  |
| Kruskal-Wallis statistic | 20.45 |  |  |  |
|  |  |  |  |  |
| Dunn's Multiple Comparison Test | Difference in rank sum | Significant? P < 0.05? | Summary |  |
| npf[LexA]/npf[1] vs w+;w1118 | 22.58 | Yes | *** |  |
| npf[LexA]/npf[1] vs npf[GR];npf[LexA] | 22.60 | Yes | *** |  |
| w+;w1118 vs npf[GR];npf[LexA] | 0.02083 | No | ns |  |

Figure 1K

|  | w+;w1118 | npf[LexA]/npf[1] | npf[GR];npf[LexA] |
| --- | --- | --- | --- |
| Number of values | 20 | 20 | 22 |
|  |  |  |  |
| 25% Percentile | 0.0 | 0.0 | 0.0 |
| Median | 0.0 | 0.1800 | 0.0 |
| 75% Percentile | 0.0425 | 0.4525 | 0.0500 |
|  |  |  |  |
| Mean | 0.0400 | 0.2295 | 0.04636 |
| Std. Deviation | 0.08473 | 0.2339 | 0.1207 |
| Std. Error | 0.01895 | 0.05230 | 0.02573 |
|  |  |  |  |
| Lower 95% CI of mean | 0.0003453 | 0.1200 | -0.007149 |
| Upper 95% CI of mean | 0.07965 | 0.3390 | 0.09988 |
|  |  |  |  |
| Sum | 0.8000 | 4.590 | 1.020 |

| Parameter |  |  |  |  |
| --- | --- | --- | --- | --- |
| Table Analyzed | MF Grouped vs decap |  |  |  |
|  |  |  |  |  |
| Kruskal-Wallis test |  |  |  |  |
| P value | 0.0061 |  |  |  |
| Exact or approximate P value? | Gaussian Approximation |  |  |  |
| P value summary | ** |  |  |  |
| Do the medians vary signif. (P < 0.05) | Yes |  |  |  |
| Number of groups | 3 |  |  |  |
| Kruskal-Wallis statistic | 10.19 |  |  |  |
|  |  |  |  |  |
| Dunn's Multiple Comparison Test | Difference in rank sum | Significant? P < 0.05? | Summary |  |
| w+;w1118 vs npf[LexA]/npf[1] | -13.55 | Yes | * |  |
| w+;w1118 vs npf[GR];npf[LexA] | 1.327 | No | ns |  |
| npf[LexA]/npf[1] vs npf[GR];npf[LexA] | 14.88 | Yes | * |  |
