# Supplementary material for: Neuropeptide F regulates courtship in Drosophila through a male-specific neuronal circuit
Source: eLife. 2019 Aug 12;8:e49574. doi: 10.7554/eLife.49574 (PMC6721794; doi:10.7554/eLife.49574)
Supplement: Figure 1—figure supplement 1—source data 2. [file elife-49574-fig1-figsupp1-data2.docx]

|  | UAS-Kir/npfG4 | UAS-DTI/npfG4 | npfG4/+ | UAS-Kir2.1/+ | UAS-DTI/+ | UAS-NaChBac/+ | UAS-NaChBac/npfG4 | UAS-npf/+ | UAS-npf/npfG4 |
| --- | --- | --- | --- | --- | --- | --- | --- | --- | --- |
| Number of values | 12 | 12 | 12 | 12 | 12 | 12 | 12 | 12 | 12 |
|  |  |  |  |  |  |  |  |  |  |
| 25% Percentile | 0.1625 | 0.1250 | 0.0 | 0.0 | 0.0 | 0.0 | 0.0 | 0.0 | 0.0 |
| Median | 0.2500 | 0.2250 | 0.0 | 0.0150 | 0.0 | 0.0 | 0.0 | 0.0 | 0.0 |
| 75% Percentile | 0.3250 | 0.2950 | 0.0450 | 0.0750 | 0.0450 | 0.0375 | 0.0475 | 0.0 | 0.0 |
|  |  |  |  |  |  |  |  |  |  |
| Mean | 0.2433 | 0.2258 | 0.02917 | 0.0375 | 0.02167 | 0.02917 | 0.02417 | 0.0100 | 0.0225 |
|  |  |  |  |  |  |  |  |  |  |
| Std. Deviation | 0.08886 | 0.09558 | 0.05401 | 0.04615 | 0.03563 | 0.06748 | 0.04033 | 0.02374 | 0.07794 |
| Std. Error | 0.02565 | 0.02759 | 0.01559 | 0.01332 | 0.01029 | 0.01948 | 0.01164 | 0.006853 | 0.0225 |
|  |  |  |  |  |  |  |  |  |  |
| Lower 95% CI of mean | 0.1869 | 0.1651 | -0.005151 | 0.008180 | -0.0009731 | -0.01371 | -0.001458 | -0.005084 | -0.02702 |
| Upper 95% CI of mean | 0.2998 | 0.2866 | 0.06348 | 0.06682 | 0.04431 | 0.07204 | 0.04979 | 0.02508 | 0.07202 |
|  |  |  |  |  |  |  |  |  |  |
| Sum | 2.920 | 2.710 | 0.3500 | 0.4500 | 0.2600 | 0.3500 | 0.2900 | 0.1200 | 0.2700 |

| Parameter |  |  |  |  |
| --- | --- | --- | --- | --- |
| Table Analyzed | npfG4 effector courtship |  |  |  |
|  |  |  |  |  |
| Kruskal-Wallis test |  |  |  |  |
| P value | < 0.0001 |  |  |  |
| Exact or approximate P value? | Gaussian Approximation |  |  |  |
| P value summary | *** |  |  |  |
| Do the medians vary signif. (P < 0.05) | Yes |  |  |  |
| Number of groups | 9 |  |  |  |
| Kruskal-Wallis statistic | 63.23 |  |  |  |
|  |  |  |  |  |
| Dunn's Multiple Comparison Test | Difference in rank sum | Significant? P < 0.05? | Summary |  |
| UAS-Kir/npfG4 vs UAS-DTI/npfG4 | 1.458 | No | ns |  |
| UAS-Kir/npfG4 vs npfG4/+ | 50.00 | Yes | *** |  |
| UAS-Kir/npfG4 vs UAS-Kir2.1/+ | 43.21 | Yes | ** |  |
| UAS-Kir/npfG4 vs UAS-DTI/+ | 51.17 | Yes | *** |  |
| UAS-Kir/npfG4 vs UAS-NaChBac/+ | 52.79 | Yes | *** |  |
| UAS-Kir/npfG4 vs UAS-NaChBac/npfG4 | 50.67 | Yes | *** |  |
| UAS-Kir/npfG4 vs UAS-npf/+ | 58.13 | Yes | *** |  |
| UAS-Kir/npfG4 vs UAS-npf/npfG4 | 58.96 | Yes | *** |  |
| UAS-DTI/npfG4 vs npfG4/+ | 48.54 | Yes | ** |  |
| UAS-DTI/npfG4 vs UAS-Kir2.1/+ | 41.75 | Yes | * |  |
| UAS-DTI/npfG4 vs UAS-DTI/+ | 49.71 | Yes | *** |  |
| UAS-DTI/npfG4 vs UAS-NaChBac/+ | 51.33 | Yes | *** |  |
| UAS-DTI/npfG4 vs UAS-NaChBac/npfG4 | 49.21 | Yes | *** |  |
| UAS-DTI/npfG4 vs UAS-npf/+ | 56.67 | Yes | *** |  |
| UAS-DTI/npfG4 vs UAS-npf/npfG4 | 57.50 | Yes | *** |  |
| npfG4/+ vs UAS-Kir2.1/+ | -6.792 | No | ns |  |
| npfG4/+ vs UAS-DTI/+ | 1.167 | No | ns |  |
| npfG4/+ vs UAS-NaChBac/+ | 2.792 | No | ns |  |
| npfG4/+ vs UAS-NaChBac/npfG4 | 0.6667 | No | ns |  |
| npfG4/+ vs UAS-npf/+ | 8.125 | No | ns |  |
| npfG4/+ vs UAS-npf/npfG4 | 8.958 | No | ns |  |
| UAS-Kir2.1/+ vs UAS-DTI/+ | 7.958 | No | ns |  |
| UAS-Kir2.1/+ vs UAS-NaChBac/+ | 9.583 | No | ns |  |
| UAS-Kir2.1/+ vs UAS-NaChBac/npfG4 | 7.458 | No | ns |  |
| UAS-Kir2.1/+ vs UAS-npf/+ | 14.92 | No | ns |  |
| UAS-Kir2.1/+ vs UAS-npf/npfG4 | 15.75 | No | ns |  |
| UAS-DTI/+ vs UAS-NaChBac/+ | 1.625 | No | ns |  |
| UAS-DTI/+ vs UAS-NaChBac/npfG4 | -0.5000 | No | ns |  |
| UAS-DTI/+ vs UAS-npf/+ | 6.958 | No | ns |  |
| UAS-DTI/+ vs UAS-npf/npfG4 | 7.792 | No | ns |  |
| UAS-NaChBac/+ vs UAS-NaChBac/npfG4 | -2.125 | No | ns |  |
| UAS-NaChBac/+ vs UAS-npf/+ | 5.333 | No | ns |  |
| UAS-NaChBac/+ vs UAS-npf/npfG4 | 6.167 | No | ns |  |
| UAS-NaChBac/npfG4 vs UAS-npf/+ | 7.458 | No | ns |  |
| UAS-NaChBac/npfG4 vs UAS-npf/npfG4 | 8.292 | No | ns |  |
| UAS-npf/+ vs UAS-npf/npfG4 | 0.8333 | No | ns |  |

| Parameter |  |
| --- | --- |
| Table Analyzed | npfG4 effector courtship |
| Column A | UAS-Kir/npfG4 |
| vs | vs |
| Column D | UAS-Kir2.1/+ |
|  |  |
| Mann Whitney test |  |
| P value | < 0.0001 |
| Exact or approximate P value? | Gaussian Approximation |
| P value summary | *** |
| Are medians signif. different? (P < 0.05) | Yes |
| One- or two-tailed P value? | Two-tailed |
| Sum of ranks in column A,D | 219.5 , 80.50 |
| Mann-Whitney U | 2.500 |

| Parameter |  |
| --- | --- |
| Table Analyzed | Data 1 |
| Column B | UAS-DTI/npfG4 |
| vs | vs |
| Column E | UAS-DTI/+ |
|  |  |
| Mann Whitney test |  |
| P value | < 0.0001 |
| Exact or approximate P value? | Gaussian Approximation |
| P value summary | *** |
| Are medians signif. different? (P < 0.05) | Yes |
| One- or two-tailed P value? | Two-tailed |
| Sum of ranks in column B,E | 222 , 78 |
| Mann-Whitney U | 0.0000 |
