# Supplementary material for: Neuropeptide F regulates courtship in Drosophila through a male-specific neuronal circuit
Source: eLife. 2019 Aug 12;8:e49574. doi: 10.7554/eLife.49574 (PMC6721794; doi:10.7554/eLife.49574)
Supplement: Figure 1—figure supplement 1—source data 4. [file elife-49574-fig1-figsupp1-data4.docx]

|  | npfG4/+ | UAS-Kir2.1/+ | UAS-Kir2.1/npfG4 | UAS-DTI/+ | UAS-DTI/npfG4 | UAS-NaChBac/+ | UAS-NaChBac/npfG4 | UAS-npf/+ | UAS-npf/npfG4 |
| --- | --- | --- | --- | --- | --- | --- | --- | --- | --- |
| Number of values | 12 | 10 | 12 | 12 | 12 | 12 | 12 | 12 | 12 |
|  |  |  |  |  |  |  |  |  |  |
| 25% Percentile | 4.250 | 1.000 | 0.0 | 0.2500 | 0.0 | 0.0 | 14.50 | 0.0 | 20.00 |
| Median | 6.500 | 5.500 | 0.0 | 4.500 | 0.0 | 0.0 | 26.00 | 3.500 | 32.50 |
| 75% Percentile | 10.50 | 9.750 | 0.0 | 6.750 | 0.0 | 8.500 | 33.75 | 7.000 | 38.00 |
|  |  |  |  |  |  |  |  |  |  |
| Mean | 7.500 | 6.000 | 0.08333 | 4.500 | 0.08333 | 3.667 | 24.92 | 4.333 | 30.75 |
| Std. Deviation | 4.123 | 5.055 | 0.2887 | 4.563 | 0.2887 | 4.887 | 10.64 | 5.033 | 10.32 |
| Std. Error | 1.190 | 1.599 | 0.08333 | 1.317 | 0.08333 | 1.411 | 3.071 | 1.453 | 2.980 |
|  |  |  |  |  |  |  |  |  |  |
| Lower 95% CI of mean | 4.880 | 2.384 | -0.1001 | 1.601 | -0.1001 | 0.5619 | 18.16 | 1.135 | 24.19 |
| Upper 95% CI of mean | 10.12 | 9.616 | 0.2667 | 7.399 | 0.2667 | 6.771 | 31.68 | 7.531 | 37.31 |
|  |  |  |  |  |  |  |  |  |  |
| Sum | 90.00 | 60.00 | 1.000 | 54.00 | 1.000 | 44.00 | 299.0 | 52.00 | 369.0 |

| Parameter |  |  |  |  |
| --- | --- | --- | --- | --- |
| Table Analyzed | npfG4_effector Aggression |  |  |  |
|  |  |  |  |  |
| Kruskal-Wallis test |  |  |  |  |
| P value | < 0.0001 |  |  |  |
| Exact or approximate P value? | Gaussian Approximation |  |  |  |
| P value summary | *** |  |  |  |
| Do the medians vary signif. (P < 0.05) | Yes |  |  |  |
| Number of groups | 9 |  |  |  |
| Kruskal-Wallis statistic | 74.89 |  |  |  |
|  |  |  |  |  |
| Dunn's Multiple Comparison Test | Difference in rank sum | Significant? P < 0.05? | Summary |  |
| npfG4/+ vs UAS-Kir2.1/+ | 6.883 | No | ns |  |
| npfG4/+ vs UAS-Kir2.1/npfG4 | 42.04 | Yes | * |  |
| npfG4/+ vs UAS-DTI/+ | 15.00 | No | ns |  |
| npfG4/+ vs UAS-DTI/npfG4 | 42.04 | Yes | * |  |
| npfG4/+ vs UAS-NaChBac/+ | 23.13 | No | ns |  |
| npfG4/+ vs UAS-NaChBac/npfG4 | -27.00 | No | ns |  |
| npfG4/+ vs UAS-npf/+ | 18.58 | No | ns |  |
| npfG4/+ vs UAS-npf/npfG4 | -32.67 | No | ns |  |
| UAS-Kir2.1/+ vs UAS-Kir2.1/npfG4 | 35.16 | No | ns |  |
| UAS-Kir2.1/+ vs UAS-DTI/+ | 8.117 | No | ns |  |
| UAS-Kir2.1/+ vs UAS-DTI/npfG4 | 35.16 | No | ns |  |
| UAS-Kir2.1/+ vs UAS-NaChBac/+ | 16.24 | No | ns |  |
| UAS-Kir2.1/+ vs UAS-NaChBac/npfG4 | -33.88 | No | ns |  |
| UAS-Kir2.1/+ vs UAS-npf/+ | 11.70 | No | ns |  |
| UAS-Kir2.1/+ vs UAS-npf/npfG4 | -39.55 | No | ns |  |
| UAS-Kir2.1/npfG4 vs UAS-DTI/+ | -27.04 | No | ns |  |
| UAS-Kir2.1/npfG4 vs UAS-DTI/npfG4 | 0.0000 | No | ns |  |
| UAS-Kir2.1/npfG4 vs UAS-NaChBac/+ | -18.92 | No | ns |  |
| UAS-Kir2.1/npfG4 vs UAS-NaChBac/npfG4 | -69.04 | Yes | *** |  |
| UAS-Kir2.1/npfG4 vs UAS-npf/+ | -23.46 | No | ns |  |
| UAS-Kir2.1/npfG4 vs UAS-npf/npfG4 | -74.71 | Yes | *** |  |
| UAS-DTI/+ vs UAS-DTI/npfG4 | 27.04 | No | ns |  |
| UAS-DTI/+ vs UAS-NaChBac/+ | 8.125 | No | ns |  |
| UAS-DTI/+ vs UAS-NaChBac/npfG4 | -42.00 | Yes | * |  |
| UAS-DTI/+ vs UAS-npf/+ | 3.583 | No | ns |  |
| UAS-DTI/+ vs UAS-npf/npfG4 | -47.67 | Yes | ** |  |
| UAS-DTI/npfG4 vs UAS-NaChBac/+ | -18.92 | No | ns |  |
| UAS-DTI/npfG4 vs UAS-NaChBac/npfG4 | -69.04 | Yes | *** |  |
| UAS-DTI/npfG4 vs UAS-npf/+ | -23.46 | No | ns |  |
| UAS-DTI/npfG4 vs UAS-npf/npfG4 | -74.71 | Yes | *** |  |
| UAS-NaChBac/+ vs UAS-NaChBac/npfG4 | -50.13 | Yes | ** |  |
| UAS-NaChBac/+ vs UAS-npf/+ | -4.542 | No | ns |  |
| UAS-NaChBac/+ vs UAS-npf/npfG4 | -55.79 | Yes | *** |  |
| UAS-NaChBac/npfG4 vs UAS-npf/+ | 45.58 | Yes | ** |  |
| UAS-NaChBac/npfG4 vs UAS-npf/npfG4 | -5.667 | No | ns |  |
| UAS-npf/+ vs UAS-npf/npfG4 | -51.25 | Yes | ** |  |

| Parameter |  |
| --- | --- |
| Table Analyzed | Data 1 |
| Column B | UAS-Kir2.1/+ |
| vs | vs |
| Column C | UAS-Kir2.1/npfG4 |
|  |  |
| Mann Whitney test |  |
| P value | 0.0002 |
| Exact or approximate P value? | Gaussian Approximation |
| P value summary | *** |
| Are medians signif. different? (P < 0.05) | Yes |
| One- or two-tailed P value? | Two-tailed |
| Sum of ranks in column B,C | 167.5 , 85.50 |
| Mann-Whitney U | 7.500 |

| Parameter |  |
| --- | --- |
| Table Analyzed | Data 1 |
| Column D | UAS-DTI/+ |
| vs | vs |
| Column E | UAS-DTI/npfG4 |
|  |  |
| Mann Whitney test |  |
| P value | 0.0009 |
| Exact or approximate P value? | Gaussian Approximation |
| P value summary | *** |
| Are medians signif. different? (P < 0.05) | Yes |
| One- or two-tailed P value? | Two-tailed |
| Sum of ranks in column D,E | 202 , 98 |
| Mann-Whitney U | 20.00 |

| Parameter |  |
| --- | --- |
| Table Analyzed | Data 1 |
| Column F | UAS-NaChBac/+ |
| vs | vs |
| Column G | UAS-NaChBac/npfG4 |
|  |  |
| Mann Whitney test |  |
| P value | 0.0001 |
| Exact or approximate P value? | Gaussian Approximation |
| P value summary | *** |
| Are medians signif. different? (P < 0.05) | Yes |
| One- or two-tailed P value? | Two-tailed |
| Sum of ranks in column F,G | 83 , 217 |
| Mann-Whitney U | 5.000 |

| Parameter |  |
| --- | --- |
| Table Analyzed | Data 1 |
| Column H | UAS-npf/+ |
| vs | vs |
| Column I | UAS-npf/npfG4 |
|  |  |
| Mann Whitney test |  |
| P value | < 0.0001 |
| Exact or approximate P value? | Gaussian Approximation |
| P value summary | *** |
| Are medians signif. different? (P < 0.05) | Yes |
| One- or two-tailed P value? | Two-tailed |
| Sum of ranks in column H,I | 78.50 , 221.5 |
| Mann-Whitney U | 0.5000 |
