# Supplementary material for: Neuropeptide F regulates courtship in Drosophila through a male-specific neuronal circuit
Source: eLife. 2019 Aug 12;8:e49574. doi: 10.7554/eLife.49574 (PMC6721794; doi:10.7554/eLife.49574)
Supplement: Figure 1—figure supplement 2—source data 2. [file elife-49574-fig1-figsupp2-data2.docx]

|  | w+;w1118 | npf[LexA] | w+;npf[1] |
| --- | --- | --- | --- |
| Number of values | 24 | 24 | 24 |
|  |  |  |  |
| 25% Percentile | 0.0 | 0.0 | 0.0 |
| Median | 0.0 | 0.2700 | 0.1700 |
| 75% Percentile | 0.1900 | 0.5650 | 0.5150 |
|  |  |  |  |
| Mean | 0.1021 | 0.3154 | 0.2671 |
| Std. Deviation | 0.1851 | 0.3255 | 0.2945 |
| Std. Error | 0.03778 | 0.06644 | 0.06011 |
|  |  |  |  |
| Lower 95% CI of mean | 0.02393 | 0.1780 | 0.1427 |
| Upper 95% CI of mean | 0.1802 | 0.4529 | 0.3914 |
|  |  |  |  |
| Sum | 2.450 | 7.570 | 6.410 |

| Parameter |  |  |  |  |
| --- | --- | --- | --- | --- |
| Table Analyzed | npf mutant MF grouped vs mature |  |  |  |
|  |  |  |  |  |
| Kruskal-Wallis test |  |  |  |  |
| P value | 0.0139 |  |  |  |
| Exact or approximate P value? | Gaussian Approximation |  |  |  |
| P value summary | * |  |  |  |
| Do the medians vary signif. (P < 0.05) | Yes |  |  |  |
| Number of groups | 3 |  |  |  |
| Kruskal-Wallis statistic | 8.545 |  |  |  |
|  |  |  |  |  |
| Dunn's Multiple Comparison Test | Difference in rank sum | Significant? P < 0.05? | Summary |  |
| w+;w1118 vs npf[LexA] | -15.29 | Yes | * |  |
| w+;w1118 vs w+;npf[1] | -13.96 | Yes | * |  |
| npf[LexA] vs w+;npf[1] | 1.333 | No | ns |  |
