# Supplementary material for: Neuropeptide F regulates courtship in Drosophila through a male-specific neuronal circuit
Source: eLife. 2019 Aug 12;8:e49574. doi: 10.7554/eLife.49574 (PMC6721794; doi:10.7554/eLife.49574)
Supplement: Figure 1—figure supplement 2—source data 4. [file elife-49574-fig1-figsupp2-data4.docx]

|  | w+;w1118 | npf[LexA] | w+;npf[1] |
| --- | --- | --- | --- |
| Number of values | 19 | 18 | 15 |
|  |  |  |  |
| 25% Percentile | 0.0 | 0.0 | 0.0 |
| Median | 0.0 | 0.1350 | 0.1200 |
| 75% Percentile | 0.0500 | 0.4425 | 0.4300 |
|  |  |  |  |
| Mean | 0.03053 | 0.2289 | 0.2033 |
| Std. Deviation | 0.05462 | 0.2528 | 0.2305 |
| Std. Error | 0.01253 | 0.05959 | 0.05951 |
|  |  |  |  |
| Lower 95% CI of mean | 0.004202 | 0.1032 | 0.07569 |
| Upper 95% CI of mean | 0.05685 | 0.3546 | 0.3310 |
|  |  |  |  |
| Sum | 0.5800 | 4.120 | 3.050 |

| Parameter |  |  |  |  |
| --- | --- | --- | --- | --- |
| Table Analyzed | npf mutant MM |  |  |  |
|  |  |  |  |  |
| Kruskal-Wallis test |  |  |  |  |
| P value | 0.0081 |  |  |  |
| Exact or approximate P value? | Gaussian Approximation |  |  |  |
| P value summary | ** |  |  |  |
| Do the medians vary signif. (P < 0.05) | Yes |  |  |  |
| Number of groups | 3 |  |  |  |
| Kruskal-Wallis statistic | 9.630 |  |  |  |
|  |  |  |  |  |
| Dunn's Multiple Comparison Test | Difference in rank sum | Significant? P < 0.05? | Summary |  |
| w+;w1118 vs npf[LexA] | -13.02 | Yes | * |  |
| w+;w1118 vs w+;npf[1] | -12.66 | Yes | * |  |
| npf[LexA] vs w+;npf[1] | 0.3611 | No | ns |  |
