# Supplementary material for: Neuropeptide F regulates courtship in Drosophila through a male-specific neuronal circuit
Source: eLife. 2019 Aug 12;8:e49574. doi: 10.7554/eLife.49574 (PMC6721794; doi:10.7554/eLife.49574)
Supplement: Figure 4—source data 2. [file elife-49574-fig4-data2.docx]

|  | npfG4 fruFLP-23 | npfG4 fruFLP-31 | UAS_stop_Shi-23 | UAS_stop_Shi-31 | UAS_Shi_stop-23 | UAS_Shi_stop-31 | G4 FLP UAS_Shi_stop-23 | G4 FLP UAS_Shi_stop-31 | G4 UAS_stop_Shi-23 | G4 UAS_stop_Shi-31 | G4 FLP UAS_stop_Shi-23 | G4 FLP UAS_stop_Shi-31 |
| --- | --- | --- | --- | --- | --- | --- | --- | --- | --- | --- | --- | --- |
| Number of values | 19 | 10 | 15 | 11 | 18 | 24 | 13 | 18 | 11 | 8 | 24 | 24 |
|  |  |  |  |  |  |  |  |  |  |  |  |  |
| 25% Percentile | 0.0 | 0.0 | 0.0 | 0.0 | 0.0 | 0.0 | 0.0 | 0.0 | 0.001111 | 0.0 | 0.0 | 0.01944 |
| Median | 0.0005556 | 0.0 | 0.003889 | 0.0050 | 0.0 | 0.0005556 | 0.002222 | 0.02139 | 0.0200 | 0.0 | 0.03444 | 0.1967 |
| 75% Percentile | 0.1005 | 0.03167 | 0.03611 | 0.02667 | 0.009444 | 0.01347 | 0.2092 | 0.09514 | 0.1194 | 0.1176 | 0.1160 | 0.9481 |
|  |  |  |  |  |  |  |  |  |  |  |  |  |
| Mean | 0.05114 | 0.04644 | 0.09956 | 0.1063 | 0.02346 | 0.06227 | 0.1030 | 0.07373 | 0.09485 | 0.1425 | 0.1213 | 0.4239 |
| Std. Deviation | 0.08982 | 0.1132 | 0.2585 | 0.2406 | 0.07124 | 0.1485 | 0.1613 | 0.1305 | 0.1869 | 0.3386 | 0.2360 | 0.4354 |
| Std. Error | 0.02061 | 0.03579 | 0.06675 | 0.07255 | 0.01679 | 0.03030 | 0.04475 | 0.03076 | 0.05636 | 0.1197 | 0.04817 | 0.08888 |
|  |  |  |  |  |  |  |  |  |  |  |  |  |
| Lower 95% CI of mean | 0.007844 | -0.03452 | -0.04361 | -0.05538 | -0.01197 | -0.0004159 | 0.005494 | 0.008836 | -0.03073 | -0.1406 | 0.02166 | 0.2400 |
| Upper 95% CI of mean | 0.09443 | 0.1274 | 0.2427 | 0.2679 | 0.05888 | 0.1250 | 0.2005 | 0.1386 | 0.2204 | 0.4256 | 0.2210 | 0.6077 |
|  |  |  |  |  |  |  |  |  |  |  |  |  |
| Sum | 0.9716 | 0.4644 | 1.493 | 1.169 | 0.4222 | 1.494 | 1.339 | 1.327 | 1.043 | 1.140 | 2.912 | 10.17 |

| Parameter |  |
| --- | --- |
| Table Analyzed | npfG4_fruFLP_Shi |
| Column K | G4 FLP UAS_stop_Shi-23 |
| vs | vs |
| Column L | G4 FLP UAS_stop_Shi-31 |
|  |  |
| Mann Whitney test |  |
| P value | 0.0056 |
| Exact or approximate P value? | Gaussian Approximation |
| P value summary | ** |
| Are medians signif. different? (P < 0.05) | Yes |
| One- or two-tailed P value? | Two-tailed |
| Sum of ranks in column K,L | 454 , 722 |
| Mann-Whitney U | 154.0 |
