# Supplementary material for: Neuropeptide F regulates courtship in Drosophila through a male-specific neuronal circuit
Source: eLife. 2019 Aug 12;8:e49574. doi: 10.7554/eLife.49574 (PMC6721794; doi:10.7554/eLife.49574)
Supplement: Figure 4—source data 4. [file elife-49574-fig4-data4.docx]

|  | npfG4 fruFLP-23 | npfG4 fruFLP-29 | UAS>stop>trpA1-23 | UAS>stop>trpA1-29 | npfG4 UAS>stop>trpA1-23 | npfG4 UAS>stop>trpA1-29 | G4 FLP UAS>stop>trpA1-23 | G4 FLP UAS>stop>trpA1-29 |
| --- | --- | --- | --- | --- | --- | --- | --- | --- |
| Number of values | 20 | 19 | 23 | 24 | 6 | 9 | 27 | 25 |
|  |  |  |  |  |  |  |  |  |
| 25% Percentile | 0.0150 | 0.001667 | 0.006667 | 0.0 | 0.01625 | 0.009167 | 0.06833 | 0.0 |
| Median | 0.1050 | 0.2467 | 0.1583 | 0.1408 | 0.3800 | 0.2883 | 0.7400 | 0.006667 |
| 75% Percentile | 0.5883 | 0.7333 | 0.7833 | 0.9450 | 0.9696 | 0.8675 | 0.9667 | 0.6767 |
|  |  |  |  |  |  |  |  |  |
| Mean | 0.2658 | 0.3761 | 0.3609 | 0.3809 | 0.4556 | 0.4104 | 0.5425 | 0.2834 |
| Std. Deviation | 0.3245 | 0.3926 | 0.3977 | 0.4397 | 0.4620 | 0.4193 | 0.4218 | 0.3637 |
| Std. Error | 0.07255 | 0.09006 | 0.08293 | 0.08975 | 0.1886 | 0.1398 | 0.08117 | 0.07273 |
|  |  |  |  |  |  |  |  |  |
| Lower 95% CI of mean | 0.1140 | 0.1868 | 0.1889 | 0.1952 | -0.02931 | 0.08807 | 0.3757 | 0.1333 |
| Upper 95% CI of mean | 0.4177 | 0.5653 | 0.5329 | 0.5666 | 0.9404 | 0.7327 | 0.7094 | 0.4335 |
|  |  |  |  |  |  |  |  |  |
| Sum | 5.317 | 7.145 | 8.300 | 9.142 | 2.733 | 3.693 | 14.65 | 7.085 |

| Parameter |  |
| --- | --- |
| Table Analyzed | npfG4_fruFLP_TrpA1 |
| Column G | G4 FLP UAS>stop>trpA1-23 |
| vs | vs |
| Column H | G4 FLP UAS>stop>trpA1-29 |
|  |  |
| Mann Whitney test |  |
| P value | 0.0066 |
| Exact or approximate P value? | Gaussian Approximation |
| P value summary | ** |
| Are medians signif. different? (P < 0.05) | Yes |
| One- or two-tailed P value? | Two-tailed |
| Sum of ranks in column G,H | 862 , 516 |
| Mann-Whitney U | 191.0 |

| Parameter |  |  |  |  |
| --- | --- | --- | --- | --- |
| Table Analyzed | npfG4_fruFLP_TrpA1 |  |  |  |
|  |  |  |  |  |
| Kruskal-Wallis test |  |  |  |  |
| P value | 0.3020 |  |  |  |
| Exact or approximate P value? | Gaussian Approximation |  |  |  |
| P value summary | ns |  |  |  |
| Do the medians vary signif. (P < 0.05) | No |  |  |  |
| Number of groups | 4 |  |  |  |
| Kruskal-Wallis statistic | 3.648 |  |  |  |
|  |  |  |  |  |
| Dunn's Multiple Comparison Test | Difference in rank sum | Significant? P < 0.05? | Summary |  |
| npfG4 fruFLP-29 vs UAS>stop>trpA1-29 | 2.348 | No | ns |  |
| npfG4 fruFLP-29 vs npfG4 UAS>stop>trpA1-29 | -4.354 | No | ns |  |
| npfG4 fruFLP-29 vs G4 FLP UAS>stop>trpA1-29 | 9.688 | No | ns |  |
| UAS>stop>trpA1-29 vs npfG4 UAS>stop>trpA1-29 | -6.701 | No | ns |  |
| UAS>stop>trpA1-29 vs G4 FLP UAS>stop>trpA1-29 | 7.341 | No | ns |  |
| npfG4 UAS>stop>trpA1-29 vs G4 FLP UAS>stop>trpA1-29 | 14.04 | No | ns |  |
