# Supplementary material for: Neuropeptide F regulates courtship in Drosophila through a male-specific neuronal circuit
Source: eLife. 2019 Aug 12;8:e49574. doi: 10.7554/eLife.49574 (PMC6721794; doi:10.7554/eLife.49574)
Supplement: Figure 4—source data 6. [file elife-49574-fig4-data6.docx]

|  | UAS-npf-RNAi/+ | elav-G4/+ | elav-G4/UAS-npf-RNAi | fru-G4/+ | fru-G4/UAS-npf-RNAi | npf-G4/+ | npf-G4/UAS-npf-RNAi | tub>stop>Gal80/+;fruFLP/+ | tub>stop>Gal80/npfG4;fruFLP/IR | tub>Gal80>stop/+;fruFLP/+ | tub>Gal80>stop/npfG4;fruFLP/IR |
| --- | --- | --- | --- | --- | --- | --- | --- | --- | --- | --- | --- |
| Number of values | 12 | 12 | 12 | 12 | 12 | 12 | 12 | 11 | 7 | 12 | 8 |
|  |  |  |  |  |  |  |  |  |  |  |  |
| Minimum | 0.0 | 0.0 | 0.0 | 0.0 | 0.0300 | 0.0 | 0.0 | 0.0 | 0.0 | 0.0 | 0.0 |
| 25% Percentile | 0.0 | 0.0 | 0.0625 | 0.0 | 0.0850 | 0.0 | 0.1500 | 0.0 | 0.0 | 0.0 | 0.1350 |
| Median | 0.0 | 0.0 | 0.2000 | 0.0 | 0.2150 | 0.0 | 0.2300 | 0.0 | 0.0 | 0.0 | 0.2650 |
| 75% Percentile | 0.0150 | 0.0 | 0.3700 | 0.0350 | 0.3450 | 0.0300 | 0.3650 | 0.0 | 0.0200 | 0.0100 | 0.4050 |
| Maximum | 0.1000 | 0.0800 | 0.5300 | 0.1000 | 0.4100 | 0.1700 | 0.4400 | 0.1200 | 0.0400 | 0.0500 | 0.5700 |
|  |  |  |  |  |  |  |  |  |  |  |  |
| Mean | 0.01417 | 0.01083 | 0.2325 | 0.0200 | 0.2183 | 0.02583 | 0.2400 | 0.01391 | 0.01029 | 0.00775 | 0.2675 |
| Std. Deviation | 0.03088 | 0.02610 | 0.1729 | 0.03516 | 0.1355 | 0.05435 | 0.1376 | 0.03655 | 0.01529 | 0.01510 | 0.1821 |
| Std. Error | 0.008915 | 0.007534 | 0.04993 | 0.01015 | 0.03912 | 0.01569 | 0.03973 | 0.01102 | 0.005781 | 0.004359 | 0.06439 |
|  |  |  |  |  |  |  |  |  |  |  |  |
| Lower 95% CI of mean | -0.005456 | -0.005748 | 0.1226 | -0.002341 | 0.1322 | -0.008698 | 0.1525 | -0.01065 | -0.003859 | -0.001844 | 0.1152 |
| Upper 95% CI of mean | 0.03379 | 0.02741 | 0.3424 | 0.04234 | 0.3044 | 0.06036 | 0.3275 | 0.03847 | 0.02443 | 0.01734 | 0.4198 |
|  |  |  |  |  |  |  |  |  |  |  |  |
| Sum | 0.1700 | 0.1300 | 2.790 | 0.2400 | 2.620 | 0.3100 | 2.880 | 0.1530 | 0.0720 | 0.09300 | 2.140 |

| Parameter |  |
| --- | --- |
| Table Analyzed | MM UAS-npf-RNAi |
| Column B | elav-G4/+ |
| vs | vs |
| Column C | elav-G4/UAS-npf-RNAi |
|  |  |
| Mann Whitney test |  |
| P value | 0.0002 |
| Exact or approximate P value? | Gaussian Approximation |
| P value summary | *** |
| Are medians signif. different? (P < 0.05) | Yes |
| One- or two-tailed P value? | Two-tailed |
| Sum of ranks in column B,C | 88 , 212 |
| Mann-Whitney U | 10.00 |

| Parameter |  |
| --- | --- |
| Table Analyzed | MM UAS-npf-RNAi |
| Column D | fru-G4/+ |
| vs | vs |
| Column E | fru-G4/UAS-npf-RNAi |
|  |  |
| Mann Whitney test |  |
| P value | 0.0001 |
| Exact or approximate P value? | Gaussian Approximation |
| P value summary | *** |
| Are medians signif. different? (P < 0.05) | Yes |
| One- or two-tailed P value? | Two-tailed |
| Sum of ranks in column D,E | 85 , 215 |
| Mann-Whitney U | 7.000 |

| Parameter |  |
| --- | --- |
| Table Analyzed | MM UAS-npf-RNAi |
| Column F | npf-G4/+ |
| vs | vs |
| Column G | npf-G4/UAS-npf-RNAi |
|  |  |
| Mann Whitney test |  |
| P value | 0.0003 |
| Exact or approximate P value? | Gaussian Approximation |
| P value summary | *** |
| Are medians signif. different? (P < 0.05) | Yes |
| One- or two-tailed P value? | Two-tailed |
| Sum of ranks in column F,G | 88.50 , 211.5 |
| Mann-Whitney U | 10.50 |

| Parameter |  |
| --- | --- |
| Table Analyzed | MM UAS-npf-RNAi |
| Column H | tub>stop>Gal80/+;fruFLP/+ |
| vs | vs |
| Column I | tub>stop>Gal80/npfG4;fruFLP/IR |
|  |  |
| Mann Whitney test |  |
| P value | 0.4224 |
| Exact or approximate P value? | Gaussian Approximation |
| P value summary | ns |
| Are medians signif. different? (P < 0.05) | No |
| One- or two-tailed P value? | Two-tailed |
| Sum of ranks in column H,I | 97 , 74 |
| Mann-Whitney U | 31.00 |

| Parameter |  |
| --- | --- |
| Table Analyzed | MM UAS-npf-RNAi |
| Column J | tub>Gal80>stop/+;fruFLP/+ |
| vs | vs |
| Column K | tub>Gal80>stop/npfG4;fruFLP/IR |
|  |  |
| Mann Whitney test |  |
| P value | 0.0014 |
| Exact or approximate P value? | Gaussian Approximation |
| P value summary | ** |
| Are medians signif. different? (P < 0.05) | Yes |
| One- or two-tailed P value? | Two-tailed |
| Sum of ranks in column J,K | 86 , 124 |
| Mann-Whitney U | 8.000 |
