# Supplementary figures and images for: Neuropeptide F regulates courtship in Drosophila through a male-specific neuronal circuit
Source: eLife. 2019 Aug 12;8:e49574. doi: 10.7554/eLife.49574 (PMC6721794; doi:10.7554/eLife.49574)

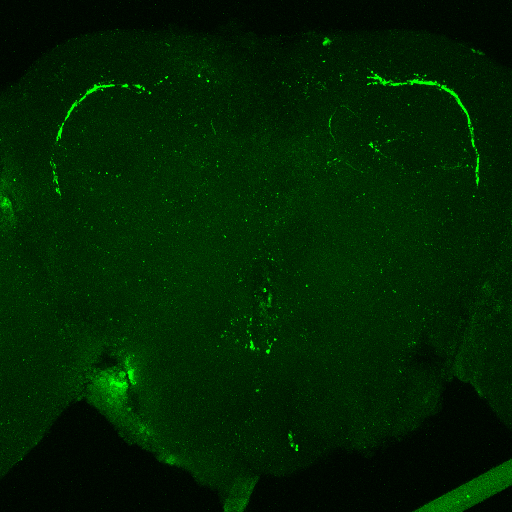

Supplement: Figure 5—source data 1. [file elife-49574-fig5-data1.zip › Figure_5H_source data_full stack.bmp]

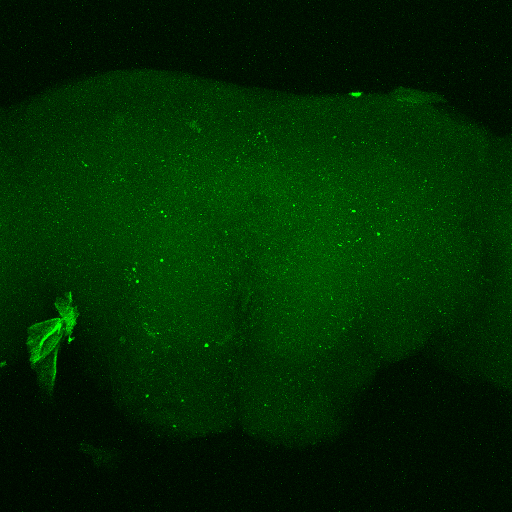

Supplement: Figure 5—source data 1. [file elife-49574-fig5-data1.zip › Figure_5I_source data_full stack.bmp]

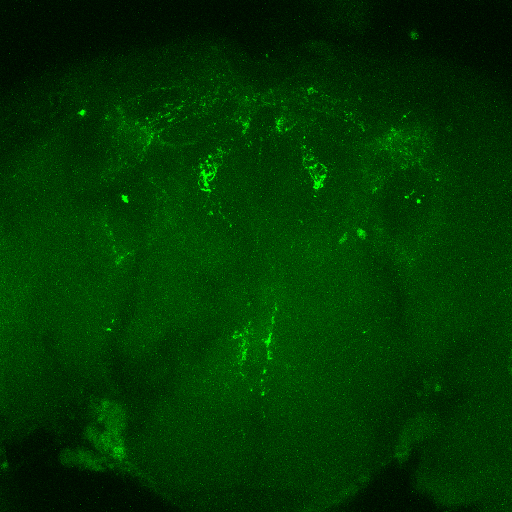

Supplement: Figure 5—source data 1. [file elife-49574-fig5-data1.zip › Figure_5A_source data_full stack.bmp]

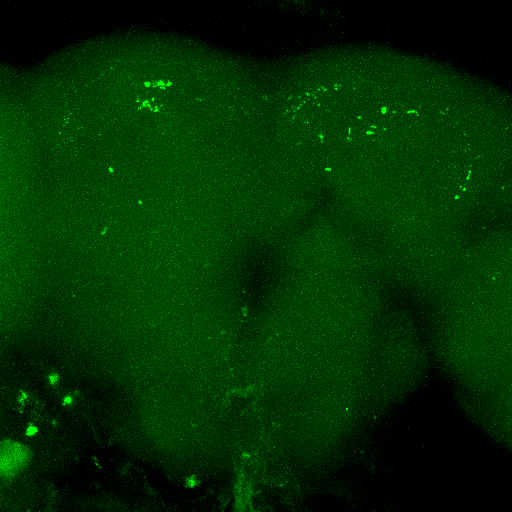

Supplement: Figure 5—source data 1. [file elife-49574-fig5-data1.zip › Figure_5B_source data_full stack.bmp]

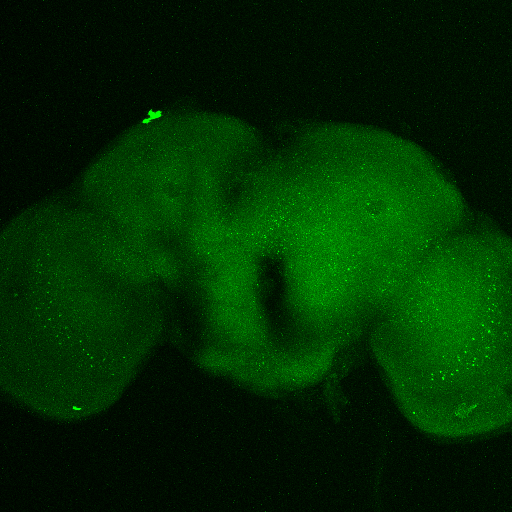

Supplement: Figure 5—source data 1. [file elife-49574-fig5-data1.zip › Figure_5C_source data_full stack.bmp]

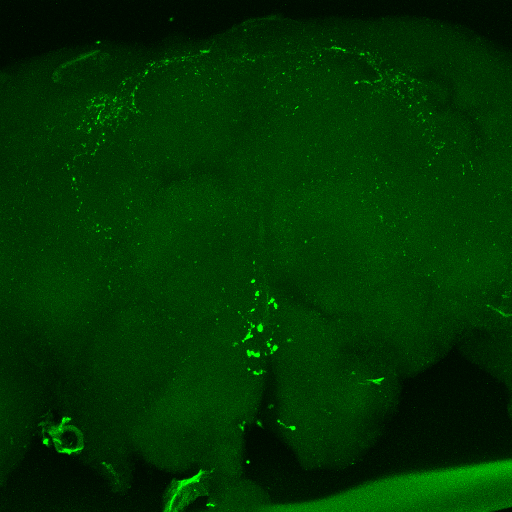

Supplement: Figure 5—source data 1. [file elife-49574-fig5-data1.zip › Figure_5G _source data_full stack.bmp]

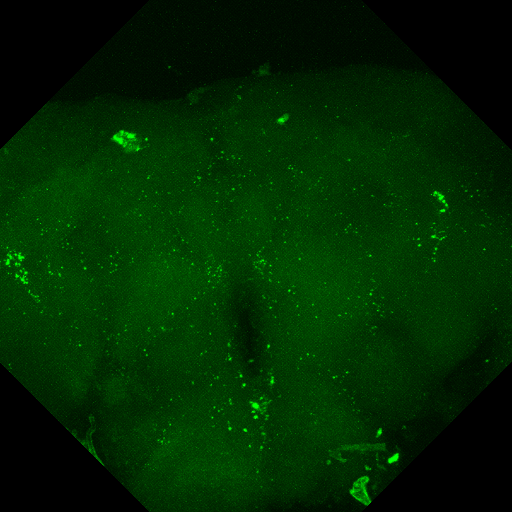

Supplement: Figure 8—source data 3. [file elife-49574-fig8-data3.zip › Figure_8A_source data_full stack.bmp]

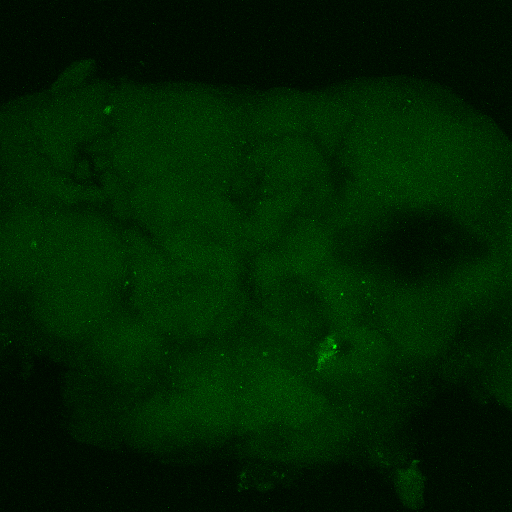

Supplement: Figure 8—source data 3. [file elife-49574-fig8-data3.zip › Figure_8B_source data_full stack.bmp]
