# Supplementary material for: Neuropeptide F regulates courtship in Drosophila through a male-specific neuronal circuit
Source: eLife. 2019 Aug 12;8:e49574. doi: 10.7554/eLife.49574 (PMC6721794; doi:10.7554/eLife.49574)
Supplement: Figure 6—source data 2. [file elife-49574-fig6-data2.docx]

|  | +>P2X2 | R71G01-LexA>P2X2 |
| --- | --- | --- |
| Number of values | 12 | 15 |
|  |  |  |
| 25% Percentile | 3.012 | 35.71 |
| Median | 5.917 | 99.92 |
| 75% Percentile | 7.740 | 194.3 |
|  |  |  |
| Mean | 10.06 | 119.0 |
| Std. Deviation | 14.38 | 93.75 |
| Std. Error | 4.151 | 24.21 |
|  |  |  |
| Lower 95% CI of mean | 0.9190 | 67.11 |
| Upper 95% CI of mean | 19.19 | 170.9 |
|  |  |  |
| Sum | 120.7 | 1785 |

| Parameter |  |
| --- | --- |
| Table Analyzed | npfG4_GCaMP opP2X2 |
| Column A | +>P2X2 |
| vs | vs |
| Column B | R71G01-LexA>P2X2 |
|  |  |
| Mann Whitney test |  |
| P value | < 0.0001 |
| Exact or approximate P value? | Gaussian Approximation |
| P value summary | *** |
| Are medians signif. different? (P < 0.05) | Yes |
| One- or two-tailed P value? | Two-tailed |
| Sum of ranks in column A,B | 86 , 292 |
| Mann-Whitney U | 8.000 |
