# Supplementary material for: Neuropeptide F regulates courtship in Drosophila through a male-specific neuronal circuit
Source: eLife. 2019 Aug 12;8:e49574. doi: 10.7554/eLife.49574 (PMC6721794; doi:10.7554/eLife.49574)
Supplement: Figure 6—source data 4. [file elife-49574-fig6-data4.docx]

|  | +>P2X2 | sp-P1-Gal4>P2X2 |
| --- | --- | --- |
| Number of values | 10 | 12 |
|  |  |  |
| Minimum | -37.21 | -4.012 |
| 25% Percentile | -10.10 | 4.358 |
| Median | -6.864 | 32.68 |
| 75% Percentile | -3.001 | 259.6 |
| Maximum | 17.91 | 323.1 |
|  |  |  |
| Mean | -7.333 | 114.7 |
| Std. Deviation | 13.84 | 130.5 |
| Std. Error | 4.377 | 37.68 |
|  |  |  |
| Lower 95% CI of mean | -17.23 | 31.79 |
| Upper 95% CI of mean | 2.568 | 197.6 |
|  |  |  |
| Sum | -73.33 | 1377 |

| Parameter |  |
| --- | --- |
| Table Analyzed | npfLexA opGCaMP |
| Column A | +>P2X2 |
| vs | vs |
| Column B | sp-P1-Gal4>P2X2 |
|  |  |
| Mann Whitney test |  |
| P value | 0.0007 |
| Exact or approximate P value? | Gaussian Approximation |
| P value summary | *** |
| Are medians signif. different? (P < 0.05) | Yes |
| One- or two-tailed P value? | Two-tailed |
| Sum of ranks in column A,B | 63 , 190 |
| Mann-Whitney U | 8.000 |
