# Supplementary material for: Neuropeptide F regulates courtship in Drosophila through a male-specific neuronal circuit
Source: eLife. 2019 Aug 12;8:e49574. doi: 10.7554/eLife.49574 (PMC6721794; doi:10.7554/eLife.49574)
Supplement: Figure 7—source data 2. [file elife-49574-fig7-data2.docx]

|  | npfG4/Shi 23c | npfG4/Shi 31c | P1-G4/Shi 23c | P1-G4/Shi 31c | npf+P1/Shi 23c | npf+P1/Shi 31c |
| --- | --- | --- | --- | --- | --- | --- |
| Number of values | 4 | 4 | 4 | 4 | 4 | 4 |
|  |  |  |  |  |  |  |
| 25% Percentile | 0.0 | 66.67 | 4.167 | 0.0 | 4.167 | 0.0 |
| Median | 0.0 | 66.67 | 16.67 | 8.333 | 16.67 | 8.333 |
| 75% Percentile | 25.00 | 79.17 | 16.67 | 29.17 | 29.17 | 41.67 |
|  |  |  |  |  |  |  |
| Mean | 8.333 | 70.83 | 12.50 | 12.50 | 16.67 | 16.67 |
| Std. Deviation | 16.67 | 8.333 | 8.333 | 15.96 | 13.61 | 23.57 |
| Std. Error | 8.333 | 4.167 | 4.167 | 7.979 | 6.804 | 11.79 |
|  |  |  |  |  |  |  |
| Lower 95% CI of mean | -18.19 | 57.57 | -0.7603 | -12.89 | -4.987 | -20.84 |
| Upper 95% CI of mean | 34.85 | 84.09 | 25.76 | 37.89 | 38.32 | 54.17 |
|  |  |  |  |  |  |  |
| Sum | 33.33 | 283.3 | 50.00 | 50.00 | 66.67 | 66.67 |

| Parameter |  |
| --- | --- |
| Table Analyzed | (P1+NPF)_Shi Percentage Initiate Courtship |
| Column A | npfG4/Shi 23c |
| vs | vs |
| Column B | npfG4/Shi 31c |
|  |  |
| Mann Whitney test |  |
| P value | 0.0265 |
| Exact or approximate P value? | Gaussian Approximation |
| P value summary | * |
| Are medians signif. different? (P < 0.05) | Yes |
| One- or two-tailed P value? | Two-tailed |
| Sum of ranks in column A,B | 10 , 26 |
| Mann-Whitney U | 0.0000 |

| Parameter |  |
| --- | --- |
| Table Analyzed | (P1+NPF)_Shi Percentage Initiate Courtship |
| Column C | P1-G4/Shi 23c |
| vs | vs |
| Column D | P1-G4/Shi 31c |
|  |  |
| Mann Whitney test |  |
| P value | 1.0000 |
| Exact or approximate P value? | Gaussian Approximation |
| P value summary | ns |
| Are medians signif. different? (P < 0.05) | No |
| One- or two-tailed P value? | Two-tailed |
| Sum of ranks in column C,D | 18.50 , 17.50 |
| Mann-Whitney U | 7.500 |

| Parameter |  |
| --- | --- |
| Table Analyzed | (P1+NPF)_Shi Percentage Initiate Courtship |
| Column E | npf+P1/Shi 23c |
| vs | vs |
| Column F | npf+P1/Shi 31c |
|  |  |
| Mann Whitney test |  |
| P value | 0.8794 |
| Exact or approximate P value? | Gaussian Approximation |
| P value summary | ns |
| Are medians signif. different? (P < 0.05) | No |
| One- or two-tailed P value? | Two-tailed |
| Sum of ranks in column E,F | 19 , 17 |
| Mann-Whitney U | 7.000 |

|  | npfG4/Shi 23c | npfG4/Shi 31c | P1-G4/Shi 23c | P1-G4/Shi 31c | npf+P1/Shi 23c | npf+P1/Shi 31c |
| --- | --- | --- | --- | --- | --- | --- |
| Number of values | 24 | 24 | 24 | 24 | 24 | 24 |
|  |  |  |  |  |  |  |
| 25% Percentile | 0.0 | 0.0 | 0.0 | 0.0 | 0.0 | 0.0 |
| Median | 0.0 | 0.06833 | 0.0 | 0.0 | 0.0 | 0.0 |
| 75% Percentile | 0.0 | 0.2025 | 0.0 | 0.0 | 0.0 | 0.0 |
|  |  |  |  |  |  |  |
| Mean | 0.007083 | 0.1886 | 0.01917 | 0.02708 | 0.01667 | 0.0100 |
| Std. Deviation | 0.02612 | 0.2907 | 0.05860 | 0.07937 | 0.05322 | 0.02588 |
| Std. Error | 0.005332 | 0.05933 | 0.01196 | 0.01620 | 0.01086 | 0.005282 |
|  |  |  |  |  |  |  |
| Lower 95% CI of mean | -0.003947 | 0.06588 | -0.005578 | -0.006432 | -0.005804 | -0.0009263 |
| Upper 95% CI of mean | 0.01811 | 0.3113 | 0.04391 | 0.06060 | 0.03914 | 0.02093 |
|  |  |  |  |  |  |  |
| Sum | 0.1700 | 4.527 | 0.4600 | 0.6500 | 0.4000 | 0.2400 |

| Parameter |  |
| --- | --- |
| Table Analyzed | (P1+NPF)_Shi Preheat 20' |
| Column A | npfG4/Shi 23c |
| vs | vs |
| Column B | npfG4/Shi 31c |
|  |  |
| Mann Whitney test |  |
| P value | < 0.0001 |
| Exact or approximate P value? | Gaussian Approximation |
| P value summary | *** |
| Are medians signif. different? (P < 0.05) | Yes |
| One- or two-tailed P value? | Two-tailed |
| Sum of ranks in column A,B | 402.5 , 773.5 |
| Mann-Whitney U | 102.5 |

| Parameter |  |
| --- | --- |
| Table Analyzed | (P1+NPF)_Shi Preheat 20' |
| Column C | P1-G4/Shi 23c |
| vs | vs |
| Column D | P1-G4/Shi 31c |
|  |  |
| Mann Whitney test |  |
| P value | 0.9428 |
| Exact or approximate P value? | Gaussian Approximation |
| P value summary | ns |
| Are medians signif. different? (P < 0.05) | No |
| One- or two-tailed P value? | Two-tailed |
| Sum of ranks in column C,D | 585.5 , 590.5 |
| Mann-Whitney U | 285.5 |

| Parameter |  |
| --- | --- |
| Table Analyzed | (P1+NPF)_Shi Preheat 20' |
| Column E | npf+P1/Shi 23c |
| vs | vs |
| Column F | npf+P1/Shi 31c |
|  |  |
| Mann Whitney test |  |
| P value | 0.9873 |
| Exact or approximate P value? | Gaussian Approximation |
| P value summary | ns |
| Are medians signif. different? (P < 0.05) | No |
| One- or two-tailed P value? | Two-tailed |
| Sum of ranks in column E,F | 589 , 587 |
| Mann-Whitney U | 287.0 |

|  | npf-GAL4/UAS-Shibire 23c | npf-GAL4/UAS-Shibire 31c | P1-Gal4/UAS-Shibire 23c | P1-Gal4/UAS-Shibire 31c | npf+P1 G4/Shibire 23c | npf+P1 G4/Shibire 31c |
| --- | --- | --- | --- | --- | --- | --- |
| Number of values | 6 | 6 | 6 | 6 | 6 | 6 |
|  |  |  |  |  |  |  |
| 25% Percentile | 0.0 | 0.2725 | 0.0 | 0.0 | 0.0 | 0.0 |
| Median | 0.0165 | 0.3150 | 0.0 | 0.0100 | 0.0 | 0.0 |
| 75% Percentile | 0.03125 | 0.3625 | 0.0325 | 0.0625 | 0.0250 | 0.0250 |
|  |  |  |  |  |  |  |
| Mean | 0.0180 | 0.3183 | 0.0150 | 0.02833 | 0.01667 | 0.0100 |
| Std. Deviation | 0.01903 | 0.05345 | 0.02811 | 0.04021 | 0.04082 | 0.01673 |
| Std. Error | 0.007767 | 0.02182 | 0.01147 | 0.01641 | 0.01667 | 0.006831 |
|  |  |  |  |  |  |  |
| Lower 95% CI of mean | -0.001967 | 0.2622 | -0.01450 | -0.01386 | -0.02618 | -0.007560 |
| Upper 95% CI of mean | 0.03797 | 0.3744 | 0.04450 | 0.07053 | 0.05951 | 0.02756 |
|  |  |  |  |  |  |  |
| Sum | 0.1080 | 1.910 | 0.0900 | 0.1700 | 0.1000 | 0.0600 |

| Parameter |  |
| --- | --- |
| Table Analyzed | (P1+NPF)_Shi Chaining |
| Column A | npf-GAL4/UAS-Shibire 23c |
| vs | vs |
| Column B | npf-GAL4/UAS-Shibire 31c |
|  |  |
| Mann Whitney test |  |
| P value | 0.0050 |
| Exact or approximate P value? | Gaussian Approximation |
| P value summary | ** |
| Are medians signif. different? (P < 0.05) | Yes |
| One- or two-tailed P value? | Two-tailed |
| Sum of ranks in column A,B | 21 , 57 |
| Mann-Whitney U | 0.0000 |

| Parameter |  |
| --- | --- |
| Table Analyzed | (P1+NPF)_Shi Chaining |
| Column C | P1-Gal4/UAS-Shibire 23c |
| vs | vs |
| Column D | P1-Gal4/UAS-Shibire 31c |
|  |  |
| Mann Whitney test |  |
| P value | 0.5914 |
| Exact or approximate P value? | Gaussian Approximation |
| P value summary | ns |
| Are medians signif. different? (P < 0.05) | No |
| One- or two-tailed P value? | Two-tailed |
| Sum of ranks in column C,D | 35.50 , 42.50 |
| Mann-Whitney U | 14.50 |

| Parameter |  |
| --- | --- |
| Table Analyzed | (P1+NPF)_Shi Chaining |
| Column E | npf+P1 G4/Shibire 23c |
| vs | vs |
| Column F | npf+P1 G4/Shibire 31c |
|  |  |
| Mann Whitney test |  |
| P value | 0.7526 |
| Exact or approximate P value? | Gaussian Approximation |
| P value summary | ns |
| Are medians signif. different? (P < 0.05) | No |
| One- or two-tailed P value? | Two-tailed |
| Sum of ranks in column E,F | 37 , 41 |
| Mann-Whitney U | 16.00 |
