# Supplementary material for: Neuropeptide F regulates courtship in Drosophila through a male-specific neuronal circuit
Source: eLife. 2019 Aug 12;8:e49574. doi: 10.7554/eLife.49574 (PMC6721794; doi:10.7554/eLife.49574)
Supplement: Figure 7—source data 4. [file elife-49574-fig7-data4.docx]

|  | WT | npfr[LexA] | npfr[LexA]/[c01896] |
| --- | --- | --- | --- |
| Number of values | 12 | 12 | 24 |
|  |  |  |  |
| 25% Percentile | 0.0 | 0.2075 | 0.0850 |
| Median | 0.0 | 0.2550 | 0.1800 |
| 75% Percentile | 0.0725 | 0.3300 | 0.3000 |
|  |  |  |  |
| Mean | 0.03583 | 0.2883 | 0.2142 |
| Std. Deviation | 0.06288 | 0.1521 | 0.1880 |
| Std. Error | 0.01815 | 0.04390 | 0.03837 |
|  |  |  |  |
| Lower 95% CI of mean | -0.004118 | 0.1917 | 0.1348 |
| Upper 95% CI of mean | 0.07578 | 0.3850 | 0.2935 |
|  |  |  |  |
| Sum | 0.4300 | 3.460 | 5.140 |

| Parameter |  |  |  |  |
| --- | --- | --- | --- | --- |
| Table Analyzed | npfr mutant |  |  |  |
|  |  |  |  |  |
| Kruskal-Wallis test |  |  |  |  |
| P value | 0.0001 |  |  |  |
| Exact or approximate P value? | Gaussian Approximation |  |  |  |
| P value summary | *** |  |  |  |
| Do the medians vary signif. (P < 0.05) | Yes |  |  |  |
| Number of groups | 3 |  |  |  |
| Kruskal-Wallis statistic | 17.70 |  |  |  |
|  |  |  |  |  |
| Dunn's Multiple Comparison Test | Difference in rank sum | Significant? P < 0.05? | Summary |  |
| WT vs npfr[LexA] | -23.13 | Yes | *** |  |
| WT vs npfr[LexA]/[c01896] | -15.52 | Yes | ** |  |
| npfr[LexA] vs npfr[LexA]/[c01896] | 7.604 | No | ns |  |

|  | npfr-IR | elav/npfr-IR | P1/npfr-IR |
| --- | --- | --- | --- |
| Number of values | 21 | 21 | 23 |
|  |  |  |  |
| 25% Percentile | 0.0 | 0.0250 | 0.0 |
| Median | 0.0 | 0.1500 | 0.0 |
| 75% Percentile | 0.0 | 0.3500 | 0.0 |
|  |  |  |  |
| Mean | 0.01714 | 0.1914 | 0.009565 |
| Std. Deviation | 0.04584 | 0.1818 | 0.02868 |
| Std. Error | 0.01000 | 0.03967 | 0.005980 |
|  |  |  |  |
| Lower 95% CI of mean | -0.003724 | 0.1087 | -0.002837 |
| Upper 95% CI of mean | 0.03801 | 0.2742 | 0.02197 |
|  |  |  |  |
| Sum | 0.3600 | 4.020 | 0.2200 |

| Parameter |  |  |  |  |
| --- | --- | --- | --- | --- |
| Table Analyzed | npfr-RNAi |  |  |  |
|  |  |  |  |  |
| Kruskal-Wallis test |  |  |  |  |
| P value | < 0.0001 |  |  |  |
| Exact or approximate P value? | Gaussian Approximation |  |  |  |
| P value summary | *** |  |  |  |
| Do the medians vary signif. (P < 0.05) | Yes |  |  |  |
| Number of groups | 3 |  |  |  |
| Kruskal-Wallis statistic | 27.42 |  |  |  |
|  |  |  |  |  |
| Dunn's Multiple Comparison Test | Difference in rank sum | Significant? P < 0.05? | Summary |  |
| npfr-IR vs elav/npfr-IR | -22.45 | Yes | *** |  |
| npfr-IR vs P1/npfr-IR | -0.02277 | No | ns |  |
| elav/npfr-IR vs P1/npfr-IR | 22.43 | Yes | *** |  |
