# Supplementary material for: Neuropeptide F regulates courtship in Drosophila through a male-specific neuronal circuit
Source: eLife. 2019 Aug 12;8:e49574. doi: 10.7554/eLife.49574 (PMC6721794; doi:10.7554/eLife.49574)
Supplement: Figure 8—source data 2. [file elife-49574-fig8-data2.docx]

|  | +>P2X2 | npfrLexA>P2X2 |
| --- | --- | --- |
| Number of values | 25 | 22 |
|  |  |  |
| 25% Percentile | 3.096 | 107.3 |
| Median | 6.193 | 154.9 |
| 75% Percentile | 11.31 | 186.4 |
|  |  |  |
| Mean | 6.938 | 149.6 |
| Std. Deviation | 5.379 | 50.42 |
| Std. Error | 1.076 | 10.75 |
|  |  |  |
| Lower 95% CI of mean | 4.718 | 127.2 |
| Upper 95% CI of mean | 9.159 | 171.9 |
|  |  |  |
| Sum | 173.5 | 3290 |

| Parameter |  |
| --- | --- |
| Table Analyzed | R71G01-Gal4_GCaMP opP2X2 |
| Column A | +>P2X2 |
| vs | vs |
| Column B | npfrLexA>P2X2 |
|  |  |
| Mann Whitney test |  |
| P value | < 0.0001 |
| Exact or approximate P value? | Gaussian Approximation |
| P value summary | *** |
| Are medians signif. different? (P < 0.05) | Yes |
| One- or two-tailed P value? | Two-tailed |
| Sum of ranks in column A,B | 325 , 803 |
| Mann-Whitney U | 0.0000 |
