# Supplementary material for: Neuropeptide F regulates courtship in Drosophila through a male-specific neuronal circuit
Source: eLife. 2019 Aug 12;8:e49574. doi: 10.7554/eLife.49574 (PMC6721794; doi:10.7554/eLife.49574)
Supplement: Supplementary file 1. [file elife-49574-supp1.docx]

| **resolvedKey Resources Table** | | | | |
| --- | --- | --- | --- | --- |
| **Reagent type (species) or resource** | **Designation** | **Source or reference** | **Identifiers** | **Additional information** |
| Gene (*D. melanogaster*) | *npf* | Flybase | FBgn0027109 |  |
| Gene (*D. melanogaster*) | *npfr* | Flybase | FBgn0037408 |  |
| Gene (*D. melanogaster*) | *npf^1^* | This paper |  | Mutant allele; Materials and Methods, “Molecular biology" subsection |
| Gene (*D. melanogaster*) | *npf^LexA^* | This paper |  | Mutant allele; Materials and Methods, “Molecular biology" subsection |
| Gene (*D. melanogaster*) | *npfr^LexA^* | This paper |  | Mutant allele; Materials and Methods, “Molecular biology" subsection |
| Gene (*D. melanogaster*) | P[*g-npf^+^*] | This paper | P[acman] CH322-163E17 | Genomic transgene; Materials and Methods, “Molecular biology" subsection |
| Genetic reagent (*D. melanogaster*) | *npf-Gal4* | Bloomington Drosophila Stock Center | BDSC Cat# 25681, RRID:BDSC_25681 BDSC Cat# 25682, RRID:BDSC_25682 |  |
| Genetic reagent (*D. melanogaster*) | *elav-Gal4* | Bloomington Drosophila Stock Center | BDSC Cat# 8765, RRID:BDSC_8765 |  |
| Genetic reagent (*D. melanogaster*) | *fru-Gal4* | Bloomington Drosophila stock center | BDSC Cat# 30027, RRID:BDSC_30027 | *NP21-Gal4* |
| Genetic reagent (*D. melanogaster*) | *R71G01-Gal4* | Bloomington Drosophila stock center | BDSC Cat# 69507, RRID:BDSC_69507 |  |
| Genetic reagent (*D. melanogaster*) | *R71G01-LexA* | Bloomington Drosophila stock center | BDSC Cat# 69507, RRID:BDSC_69507 |  |
| Genetic reagent (*D. melanogaster*) | *UAS-NaChBac* | Bloomington Drosophila stock center | BDSC Cat# 9468, RRID:BDSC_9468 |  |
| Genetic reagent (*D. melanogaster*) | *UAS-Kir2.1* | Bloomington Drosophila stock center | BDSC Cat# 6596, RRID:BDSC_6596 |  |
| Genetic reagent (*D. melanogaster*) | *UAS-DTI* | Bloomington Drosophila stock center | BDSC Cat# 25039, RRID:BDSC_25039 |  |
| Genetic reagent (*D. melanogaster*) | *UAS-mCD8::GFP* | Bloomington Drosophila stock center | BDSC Cat# 5137, RRID:BDSC_5137 |  |
| Genetic reagent (*D. melanogaster*) | *UAS-npf-RNAi* | The Vienna Drosophila Resource Center | FlyBase Cat# FBst0481395, RRID:FlyBase_FBst0481395 |  |
| Genetic reagent (*D. melanogaster*) | *UAS-npfr-RNAi* | The Vienna Drosophila Resource Center | FlyBase Cat# FBst0481454, RRID:FlyBase_FBst0481454 |  |
| Genetic reagent (*D. melanogaster*) | *UAS-DenMark, UAS-Syt::eGFP* | Bloomington Drosophila stock center | BDSC Cat# 33064, RRID:BDSC_33064 |  |
| Genetic reagent (*D. melanogaster*) | *LexAop-mCherry* | Bloomington Drosophila stock center | BDSC Cat# 52271, RRID:BDSC_52271 |  |
| Genetic reagent (*D. melanogaster*) | *LexAop(FRT.mCherry)ReaChR-mCitrine* | Bloomington Drosophila stock center | BDSC Cat# 53744, RRID:BDSC_53744 |  |
| Genetic reagent (*D. melanogaster*) | *UAS-IVS-mCD8::RFP*, *LexAop-mCD8::GFP* | Bloomington Drosophila stock center | BDSC Cat# 32229, RRID:BDSC_32229 |  |
| Genetic reagent (*D. melanogaster*) | *UAS-CD4-spGFP1-10*,*LexAop-CD4-spGFP11* | Bloomington Drosophila stock center | BDSC Cat# 58755, RRID:BDSC_58755 |  |
| Genetic reagent (*D. melanogaster*) | *LexAop-IVS-CsChrimson.mVenus* | Bloomington Drosophila stock center | BDSC Cat# 55139, RRID:BDSC_55139 |  |
| Genetic reagent (*D. melanogaster*) | *Lexop*(*FRT.stop*)*myr::smGdP-V5* | Bloomington Drosophila stock center | BDSC Cat# 62107, RRID:BDSC_62107 |  |
| Genetic reagent (*D. melanogaster*) | *npfr^c01896^* | Bloomington Drosophila stock center | BDSC Cat# 10747, RRID:BDSC_10747 |  |
| Genetic reagent (*D. melanogaster*) | *tub(FRT.Gal80)stop* | Bloomington Drosophila stock center | BDSC Cat# 38880, RRID:BDSC_38880 |  |
| Genetic reagent (*D. melanogaster*) | *tub(FRT.stop)Gal80* | Bloomington Drosophila stock center | BDSC Cat# 38878, RRID:BDSC_38878 |  |
| Genetic reagent (*D. melanogaster*) | *UAS-npf* | Gift from Dr. Ping Shen |  |  |
| Genetic reagent (*D. melanogaster*) | *UAS-P2X2*,*LexAop-GCaMP3* | Gift from Dr. Orie Shafer |  |  |
| Genetic reagent (*D. melanogaster*) | *UAS-GCaMP3*,*LexAop-P2X2* | Gift from Dr. Orie Shafer |  |  |
| Genetic reagent (*D. melanogaster*) | *UAS-Shibire^ts^* | Gift from Dr. Christopher Potter |  |  |
| Genetic reagent (*D. melanogaster*) | *fru^FLP^* | Gift from Dr. Barry Dickson |  |  |
| Genetic reagent (*D. melanogaster*) | *UAS-*(*FRT.stop*)*mCD8::GFP* | Gift from Dr. Barry Dickson |  |  |
| Genetic reagent (*D. melanogaster*) | *UAS-*(*FRT.stop*)*Shibire^ts^* | Gift from Dr. Barry Dickson |  |  |
| Genetic reagent (*D. melanogaster*) | *UAS-*(*FRT.Shibire^ts^*)*stop* | Gift from Dr. Barry Dickson |  |  |
| Genetic reagent (*D. melanogaster*) | *UAS-*(*FRT.stop*)*dTRPA1* | Gift from Dr. Barry Dickson |  |  |
| Genetic reagent (*D. melanogaster*) | *R71G01-DBD*;*R15A01-AD* | Gift from Dr. David Anderson |  | *sp-P1-Gal4* |
| Genetic reagent (*D. melanogaster*) | *w^1118^* | Kept in lab stock |  |  |
| Genetic reagent (*D. melanogaster*) | *CS* | Kept in lab stock |  |  |
| Antibody | anti-GFP (chicken polyclonal) | Invitrogen | Thermo Fisher Scientific Cat# A10262, RRID:AB_2534023 | IHC (1:1000) |
| Antibody | anti-DsRed (rabbit polyclonal) | Clontech | Takara Bio Cat# 632496, RRID:AB_10013483 | IHC (1:1000) |
| Antibody | anti-Brp, termed nc82 (mouse monoclonal) | Developmental Studies Hybridoma Bank | DSHB Cat# nc82, RRID:AB_2314866 | IHC (1:250) |
| Antibody | Anti-fruM (rabbit polyclonal) | Gift from Dr. Barry Dickson |  | IHC (1:10000) |
| Antibody | Anti-dsxM (rat polyclonal) | Gift from Dr. Brian Oliver |  | IHC (1:500) |
| Antibody | Anti-NPF (rabbit polyclonal) | RayBiotech | ABIN641365 | IHC (1:500) |
| Antibody | Anti-V5 (mouse monoclonal, DyLight 549 tagged) | BioRad | Bio-Rad Cat# MCA2894D549GA, RRID:AB_10845946 | IHC (1:500) |
| Antibody | AlexaFluor 488 goat anti-chicken IgY (H+L) secondary antibody | Invitrogen | Thermo Fisher Scientific Cat# A-11039, RRID:AB_2534096 | IHC (1:1000) |
| Antibody | AlexaFluor 488 goat anti-rat IgG (H+L) secondary antibody | Invitrogen | Thermo Fisher Scientific Cat# A-11006, RRID:AB_2534074 | IHC (1:1000) |
| Antibody | AlexaFluor 568 goat anti-rabbit IgG (H+L) secondary antibody | Invitrogen | Thermo Fisher Scientific Cat# A-11011, RRID:AB_143157 | IHC (1:1000) |
| Antibody | AlexaFluor 633 goat anti-mouse IgG (H+L) secondary antibody | Invitrogen | Thermo Fisher Scientific Cat# A-21050, RRID:AB_2535718 | IHC (1:1000) |
| Antibody | Rhodamine Red-X goat anti rabbit IgG (H+L) secondary antibody | Molecular Probe | Innovative Research Cat# R6394, RRID:AB_1500693 | IHC (1:1000) |
| Recombinant DNA reagent | pU6-BbsI-ChiRNA | Addgene | RRID:Addgene_45946 |  |
| Recombinant DNA reagent | pBPLexA::p65Uw | Addgene | RRID:Addgene_26231 |  |
| Other | BestGene | <https://www.thebestgene.com/> |  | *Drosophila* transgene service; CRISPR, P[acman] injection |
| Commercial assay, kit | In-Fusion HD Cloning | Clontech | Clontech:639647 |  |
| Chemical compound, drug | ATP | Sigma | A2383-5G |  |
| Software, algorithm | Prism 5.0 | GraphPad | GraphPad Prism, RRID:SCR_002798 |  |
| Software, algorithm | ImageJ | <https://imagej.net/> | ImageJ, RRID:SCR_003070 |  |
| Software, algorithm | CRISPR Optimal Target Finder | <http://tools.flycrispr.molbio.wisc.edu/targetFinder/> |  |  |
