# Supplementary material for: Neuropeptide F regulates courtship in Drosophila through a male-specific neuronal circuit
Source: eLife. 2019 Aug 12;8:e49574. doi: 10.7554/eLife.49574 (PMC6721794; doi:10.7554/eLife.49574)
Supplement: Supplementary file 2. [file elife-49574-supp2.docx]

Supplementary Table 1

| **Figure** | **Genotype** |
| --- | --- |
| Figure 1A-C | *npf-Gal4*/+ |
| Figure 1A-C | *UAS-Shibire^ts^*/+ |
| Figure 1A-C | *npf-Gal4*/+;*UAS-Shibire^ts^*/+ |
| Figure 1F-K | *w^1118-CS^* |
| Figure 1F-K | *npf^LexA^*/*npf^1^* |
| Figure 1F-K | P[*g*-*npf^+^*]/P[*g*-*npf^+^*];*npf^LexA^*/*npf^LexA^* |
| Figure 2A-D | *npf^LexA^*/*LexAop-IVS-CsChrimson.mVenus* |
| Figure 2E | *fru^FLP^*/+ |
| Figure 2E | *fru^FLP^*/*fru^FLP^* |
| Figure 3E-J | *LexAop*(*FRT.mCherry*)*ReaChR.mCitrine*/+;*npf^LexA^*/*fru^FLP^* |
| Figure 4A | *npf-Gal4/+*;*fru^FLP^*/*+* |
| Figure 4A | *UAS>stop>Shibire^ts^*/+ |
| Figure 4A | *UAS>Shibire^ts^>stop*/+ |
| Figure 4A | *npf-Gal4*/*UAS>stop>Shibire^ts^;fru^FLP^*/*+* |
| Figure 4A | *npf-Gal4*/*UAS>Shibire^ts^>stop* |
| Figure 4A | *npf-Gal4*/*UAS>Shibire^ts^>stop;fru^FLP^*/*+* |
| Figure 4B | *npf-Gal4*/*+*;*fru^FLP^*/*+* |
| Figure 4B | *UAS>stop>dTrpA1*/+ |
| Figure 4B | *npf-Gal4*/*UAS>stop>dTrpA1* |
| Figure 4B | *npf-Gal4*/*UAS>stop>dTrpA1;fru^FLP^*/*+* |
| Figure 4C-E | *npf-Gal4*/+ |
| Figure 4C-E | *UAS-npf-RNAi*/+ |
| Figure 4C-E | *npf-Gal4*/+;*UAS-npf-RNAi*/+ |
| Figure 4F | *UAS-npf-RNAi*/+ |
| Figure 4F | *elav-Gal4*/+ |
| Figure 4F | *npf-Gal4*/+ |
| Figure 4F | *NP21-Gal4*/+ |
| Figure 4F | *elav-Gal4*/+;*UAS-npf-RNAi*/+ |
| Figure 4F | *npf-Gal4*/+;*UAS-npf-RNAi*/+ |
| Figure 4F | *NP21-Gal4*/*UAS-npf-RNAi* |
| Figure 4F | *tub>stop>Gal80*/+;*fru^FLP^*/+ |
| Figure 4F | *tub>Gal80>stop*/+;*fru^FLP^*/+ |
| Figure 4F | *npf-Gal4/tub>stop>Gal80*;*fru^FLP^*/*UAS-npf-RNAi* |
| Figure 4F | *npf-Gal4/tub>Gal80>stop*;*fru^FLP^*/*UAS-npf-RNAi* |
| Figure 5A-B | *UAS-CD4-spGFP1-10*,*lexAop-CD4-spGFP11*/*NP21-Gal4*,*npf^LexA^* |
| Figure 5C | *UAS-CD4-spGFP1-10*,*lexAop-CD4-spGFP11*/*NP21-Gal4* |
| Figure 5D-F | *UAS*(*FRT.STOP*)*mCD8::GFP*/*LexAop2*(*FRT.STOP*)*myr::smGdP-V5*;*R71G01-Gal4*,*npf^LexA^*/*fru^FLP^* |
| Figure 5G-H | *UAS-CD4-spGFP1-10*,*lexAop-CD4-spGFP11*/*R71G01-Gal4*,*npf^LexA^* |
| Figure 5I | *UAS-CD4-spGFP1-10*,*lexAop-CD4-spGFP11*/*R71G01-Gal4* |
| Figure 6A-C | *UAS-GCaMP3*,*LexAopP2X2*/*R71G01-LexA*;*npf-Gal4*/*+* |
| Figure 6A-C | *npf-Gal4*/+;*UAS-GCaMP3*,*LexAopP2X2*/+ |
| Figure 6D-F | *UAS-P2X2*,*LexAop-GCaMP3*/*R15A01-AD*;*npf^LexA^*/*R71G01-DBD* |
| Figure 6D-F | *UAS-P2X2,LexAop-GCaMP3*/+;*npf^LexA^*/+ |
| Figure 7A-C | *npf-Gal4*/+;*UAS-Shibire^ts^*/+ |
| Figure 7A-C | *R71G01-Gal4*/*UAS-Shibire^ts^* |
| Figure 7A-C | *npf-Gal4*/+;*R71G01-Gal4*/*UAS-Shibire^ts^* |
| Figure 7D-F | *UAS-mCD8::GFP.L*,*UAS-mCD8::GFP.L*/+;*R71G01-Gal4*/*npfr^LexA^*,*LexAop2-6XmCherry-HA* |
| Figure 7G | *w^1118-CS^* |
| Figure 7G | *npfr^LexA^*/*npfr^LexA^* |
| Figure 7G | *npfr^LexA^*/*npfr^c01896^* |
| Figure 7H | *UAS-npfr-RNA-RNAi*/+ |
| Figure 7H | *elav-Gal4*/*UAS-npfr-RNA-RNAi* |
| Figure 7H | *UAS-npfr-RNA-RNAi*/+;*R71G01-Gal4*/+ |
| Figure 8A and B | *UAS-spGFP1-10*,*LexAop-spGFP11*/*R71G01-Gal4*,*npfr^LexA^* |
| Figure 8C-E | *UAS-GCaMP3*,*LexAopP2X2*/+;*R71G01-Gal4*/*npfr^LexA^* |
| Figure 8C-E | *UAS-GCaMP3*,*LexAopP2X2*/+;*R71G01-Gal4*/+ |
|  |  |
| Figure 1-figure supplement 1 | *npf-Gal4*/+ |
| Figure 1-figure supplement 1 | *UAS-Kir2.1*/+ |
| Figure 1-figure supplement 1 | *UAS-DTI*/+ |
| Figure 1-figure supplement 1 | *UAS-NaChBac*/+ |
| Figure 1-figure supplement 1 | *UAS-npf-cDNA*/+ |
| Figure 1-figure supplement 1 | *npf-Gal4*/*UAS-Kir2.1* |
| Figure 1-figure supplement 1 | *npf-Gal4*/*UAS-DTI* |
| Figure 1-figure supplement 1 | *npf-Gal4*/*UAS-NaChBac* |
| Figure 1-figure supplement 1 | *npf-Gal4*/*UAS-npf-cDNA* |
| Figure 1-figure supplement 2B | *w^1118^* |
| Figure 1-figure supplement 2C | *npf^LexA^*/*npf^LexA^* |
| Figure 1-figure supplement 2D | P[*g*-*npf^+^*]/P[*g*-*npf^+^*];*npf^LexA^*/*npf^LexA^* |
| Figure 1-figure supplement 2E | *npf^1^*/*npf^1^* |
| Figure 1-figure supplement 2F and G | *w^1118-CS^* |
| Figure 1-figure supplement 2F and G | *npf^LexA^*/*npf^LexA^* |
| Figure 1-figure supplement 2F and G | *npf^1^*/*npf^1^* |
| Figure 2-figure supplement 1 | *w^1118^* |
| Figure 5-figure supplement 2 | *UAS-IVS-mCD8::RFP*,*LexAop2-mCD8::GFP*/+;*npf^LexA^*/*R71G01-Gal4* |
| Figure 5-figure supplement 3A | *UAS-DenMark*,*UAS-syt::eGFP*/+;*R71G01-Gal4*/+ |
| Figure 5-figure supplement 3B | *npf-Gal4*/*UAS-DenMark*,*UAS-syt::GFP* |
| Figure 5-figure supplement 3C | *npf-Gal4*/*UAS*(*FRT.STOP*)*mCD8::GFP*;*fru^FLP^*/+ |
| Figure 6-figure supplement 1 | *UAS-P2X2*,*LexAopGCaMP3*/+;*R71G01-Gal4*,*npf^LexA^*/+ |
|  |  |
| Video 1 | *LexAop*(*FRT.mCherry*)*mCitrine*/+;*npf^LexA^*/*fru^FLP^* |
| Video 2 | *UAS*(*FRT.stop*)*mCD8::GFP*/*LexAop*(*FRT.stop*)*myr::smGdP-V5*;*R71G01-Gal4*,*npf^LexA^*/*fru^FLP^* |
| Video 3 | *UAS-GCaMP3*,*LexAopP2X2*/*R71G01-LexA*;*npf-Gal4*/+ |
| Video 4 | *UAS-P2X2*,*LexAopGCaMP3*/+;*R71G01-Gal4,npf^LexA^*/+ |
| Video 5 | *UAS-GCaMP3*,*LexAopP2X2*/+;*R71G01-Gal4*/*npfr^LexA^* |
